# Health behaviour interventions to improve mental health outcomes for students in the university setting: a systematic review of randomised controlled trials

**DOI:** 10.1186/s12966-025-01718-7

**Published:** 2025-03-11

**Authors:** Sandya Streram, Tracy Burrows, Mitch J. Duncan, Melinda Hutchesson

**Affiliations:** 1 School of Health Sciences, College of Health, Medicine and Wellbeing, University of Newcastle, Callaghan, NSW Australia; 2 Food and Nutrition Research Program, Hunter Medical Research Institute, New Lambton Heights, NSW Australia; 3 School of Medicine and Public Health, College of Health, Medicine and Wellbeing, University of Newcastle, Callaghan, NSW Australia

**Keywords:** University student, Health behaviour (MeSH), Mental health, Systematic review, Sleep (MeSH), Exercise (MeSH), Diet, Sedentary behaviour (MeSH)

## Abstract

**Background:**

University students incur significantly elevated levels of stress compared to the general population and their non-student counterparts. Health risk behaviours are important modifiable determinants for the onset and aggravation of various mental health disorders, in which, university students generally exhibit poor engagement. Thus, this study aims to determine the efficacy of health behaviour interventions in relation to change in health behaviour and mental health outcomes, the impact of interventions (i.e., penetration, fidelity, and implementation), intervention characteristics associated with improved outcomes (efficacy) and the economic evaluation of interventions.

**Methods:**

Six electronic databases were searched for randomised controlled trials (RCT) published from the 1st January 2012 to 11th July 2023. Eligible RCTs included university students, evaluated behavioural interventions targeting health behaviours (i.e. dietary intake, physical activity, sedentary behaviour, alcohol use, substance use, smoking, and sleep) and reported a change in both health behaviour and mental health outcomes.

**Results:**

Twenty-two RCTs met the study inclusion criteria. Overall, only seven studies were effective in improving both health behaviour and mental health outcomes, with most (*n* = 4) focused on improving sleep behaviours. Insufficient evidence was found regarding intervention impact, intervention characteristics associated with improved outcomes and the economic evaluation of interventions to guide future implementation of health behaviour interventions in universities due to inadequate reporting of outcomes.

**Conclusions:**

There is limited evidence regarding the efficacy of health behaviour interventions in improving both health behaviour and mental health outcomes. There is also insufficient evidence regarding intervention impact, intervention characteristics associated with improved outcomes and economic evaluation to guide the implementation of these interventions in the university setting.

**Supplementary Information:**

The online version contains supplementary material available at 10.1186/s12966-025-01718-7.

## Background

University students incur significantly elevated levels of stress compared to the general population and their non-student counterparts [1, 2]. A 2018 global survey of 13,984 students conducted across 8 countries found that approximately one-third of students screened positive for at least one anxiety, mood, or substance disorder [3]. University commencement is characterised by unique stressors including changes in living arrangements, geographical separation from families, newfound financial and academic responsibilities, establishment of new social relationships and increased freedom over health and lifestyle choices [2, 4]. This shift towards increased autonomy and financial management may contribute to and result in the development of anxiety, depression, psychological distress, isolation, and a reduction in self–esteem among university students [2, 5, 6]. These issues are compounded by performance expectations, high levels of competition, poor diet, lack of sleep and drug and alcohol abuse, leading to early course exit [2, 7]. Concurrently, university students exhibit increased susceptibility to mental illness as the age of onset for various mental health disorders often coincides with engagement in university study [8, 9]. Thus, universities are a critical setting for the delivery of initiatives to alter student mental health trajectories.

Health risk behaviours have been identified as modifiable determinants for the onset and aggravation of various mental health disorders [10–12]. The relationship between mental health and health risk behaviours is bidirectional in nature. Mental health disorders are associated with increased engagement in health risk behaviours including substance use, physical inactivity and poor dietary habits as maladaptive coping strategies to manage negative affect [13]. However, these coping mechanisms can in turn result in increased inflammation, anxiety and other stress indicators, precipitating the occurrence of mental health conditions [14–16]. Among university students, multiple studies have demonstrated high engagement and co-occurrence of health risk behaviours which have been associated with mental health outcomes [17–23]. For example, students classified as engaging in health risk behaviours have higher likelihood of experiencing moderate and high/very high psychological distress [17, 18] Therefore, the role of health risk behaviours in the prevention and treatment of mental health disorders is proving increasingly important as an effective solution given the rising burden of mental disorders and limitations of existing approaches [11, 12].

The importance of health risk behaviours is emphasised by several leading authorities endorsing lifestyle-based approaches for the management of mood disorders as a first line of treatment [10, 24]. Existing reviews have elucidated the efficacy of health behaviour interventions for improving mental health outcomes, however, these reviews have predominantly focused on children or the general adult population [25–27]. These reviews also did not assess key components of intervention feasibility including intervention impact and economic evaluation. To ensure effective translation of evidence to practice, insight into intervention feasibility is imperative to maximise utilization of resources, enhance methodological rigor, measure implementation strategy effects and determine causal mechanisms [28]. No systematic review has been conducted to explore the efficacy of health behaviour interventions in improving mental health outcomes specifically for students in the university setting. However, a scoping review conducted to describe the extent and range of randomised controlled trials (RCTs) evaluating health behaviour interventions that measure student mental health outcomes has identified a sufficient number of RCTs in this research area, thus there is an opportunity to utilise existing evidence [29]. Therefore, the primary aim of this systematic review is to determine from RCTs, the efficacy of interventions in relation to change in health behaviour and mental health outcomes. The secondary aims of this review are to determine the impact of interventions, intervention characteristics associated with improved outcomes and the economic evaluation of interventions.

## Methods

### Protocol and registration

The conduct of this systematic review adheres to the Preferred Reporting Items for Systematic Reviews and Meta-Analyses (PRISMA) 2020 guidelines [30], with the protocol registered on Prospero [29]. The full review aimed to synthesize evidence on all health behaviour interventions delivered for students in the university setting, however, this review focuses solely on studies that evaluated health behaviour interventions, and reported both health behaviour and mental health outcomes.

### Search strategy

Six electronic databases were searched: Medline, Embase, CINAHL, Cochrane, ERCI, Education Complete, PsycINFO and Scopus. The search was limited to peer-review manuscripts, with human subjects, published in English from the 1st January 2012 to 11th July 2023. Restricting studies to this time period allowed the review to provide a contemporary evaluation of health behaviour interventions for students in the university setting, to best inform their implementation by universities. The search strategy was developed in collaboration with a senior librarian to include appropriate search terms for each health behaviour. The search was then executed systematically and adjusted for each database. The search strategy can be found in Table A1. Additionally, forward, and backward citation searching for all included studies was conducted.

### Study selection

Study selection was managed utilising Covidence software. Title, abstracts, and keywords of identified articled were assessed for eligibility by two independent reviewers (SS and MH or TB or MW or HB). Potentially eligible full texts were retrieved and screened by two independent reviewers (SS and MH), with disagreements in assessments being resolved by a third reviewer (TB). The reason for exclusion was recorded.

### Eligibility criteria

Studies were eligible for inclusion if they met the following inclusion criteria. A detailed inclusion criteria can be found in Table 1.

**Table 1:** Eligibility criteria (PICOS)

Participants and Population	University students enrolled in a tertiary education institution, namely a ‘university’ or ‘college’.
*Intervention*	Behavioural interventions implemented within a university setting, targeting one or more health behaviours of interest (i.e., dietary intake, physical activity, sedentary behaviour, alcohol intake, sleep, smoking status, or drug use) were included.
*Comparator*	Any comparator/control was considered for inclusion.
*Outcomes*	Studies reporting change in both health behaviours and mental health outcomes were included: • Dietary intake: Changes in energy, macro/micronutrients, food group intake, diet quality, and dietary patterns • Physical activity: Changes in total energy expenditure, frequency and duration of physical activity • Sedentary behaviour: Changes in frequency and duration of sitting time or recreational screen time • Alcohol intake: Changes in frequency and quantity of alcohol intake • Sleep: Sleep duration, quality, timing and alertness • Smoking status: Changes in tobacco smoking, or e-cigarette use • Drug use: Changes in frequency and quantity of use of illegal drugs, misuse or non-medical use of pharmaceutical drugs, or inappropriate use of other substances such as inhalants • Mental health: Psychological wellbeing (i.e. Changes in hedonic (e.g., happiness, positive emotions) and/or eudemonic (e.g., self-acceptance, autonomy) domains of wellbeing) and mental illness/ mental health disorder (i.e. Changes in symptoms/severity/diagnosis of all psychiatric disorders as per DSM-IV-TR)
*Study Design*	Randomized controlled trials (RCTs) including feasibility and pilot RCTs. Quasi and pseudo RCTs were excluded.

#### Participants and population

Participants were any university students enrolled in a tertiary education institution.

#### Intervention

Behavioural interventions implemented within a university setting and targeting one or more health behaviours of interest (i.e., dietary intake, physical activity, sedentary behaviour, alcohol intake, sleep, smoking status, or substance use) were included.

#### Comparator

Any comparator/control was considered for inclusion.

#### Outcomes

Studies reporting change in both health behaviours and mental health outcomes were included.

#### Study design

Only RCTs were included, including feasibility and pilot RCTs. Quasi and pseudo RCTs were excluded from this review.

### Risk of bias assessment

Risk of bias for eligible studies were assessed using the Cochrane Collaboration Tool [31] which considers sequence generation, allocation concealment, blinding of participants/personnel, blinding of outcome assessment, incomplete outcome data, selective outcome reporting and other undefined sources of bias. Each criterion was rated yes/low risk of bias, no/high risk of bias or unclear by two independent reviewers (SS and KS), with disagreements being resolved by in assessments being resolved by a third reviewer (MH).

### Data extraction and synthesis

Data extraction was completed by one reviewer (SS) and checked by a second reviewer (KS) using a standardized data extraction tool developed by the authors. The extracted data included study characteristics (e.g. country, year of publication, health behaviour-related inclusion criteria, student-related inclusion criteria, mental health-related inclusion criteria) and sample characteristics (e.g. number, age, sex). Data was extracted to assess each aim of this systematic review as described in Table 2.

**Table 2:** Data extraction and synthesis to assess systematic review aims

Aims	Data Extraction	Data Synthesis
Intervention efficacy	Type of health behaviour and mental health outcome measures, measurement tools used, measurement timepoints and significance of findings were extracted to determine intervention efficacy.	An intervention was deemed effective if it reported a statistically significant improvement in both health behaviour outcome and mental health outcome.
Intervention impact	The PIPE Impact Metric [67] was utilised to assess intervention impact (penetration, implementation, participation and effect). • Penetration: the proportion of the target group reached by invitations to engage in the study. • Implementation: the degree to which the intervention was implemented according to plan (i.e. fidelity). Studies were classified as having low fidelity if no measures of fidelity were reported (e.g., manual, checklist, quality measures including session recordings), moderate fidelity if a manual but no checklist or quality measures were reported and high fidelity if both a manual and checklist or quality measures were reported. • Participation: the proportion of invited individuals who enrolled in the study and effect was defined as a statistically significant improvement in both health behaviour and mental health outcomes.	
Intervention characteristics associated with improved outcomes	Intervention characteristics were assessed according to the Template for Intervention Description and Replication (TIDieR) checklist [68]. Data was extracted to describe the Why (theoretical framework), Who (intervention provider), Where (intervention location), How (mode of delivery and delivery format), When (intervention duration) and How much (number of sessions), Tailoring (e.g. interventions that were personalized), Modification (e.g. interventions that were modified during course of the study) and How Well (retention rates and fidelity). Data collected on intervention provider was classified as health professional, peer, automated delivery or other. Data collected on intervention location was classified as researcher-based (i.e. participant goes to the intervention e.g., at research centre, health care centre etc. within the university), participant-based (i.e. intervention comes to the participant e.g. within their dorm, home etc.) or a combination of both participant-based and research-based. Data collected on mode of delivery was classified as in person, telephone/telehealth or technology-based (excluding telephone/telehealth) while data on delivery format was classified as individual, group or both. The total number of sessions (how much) were calculated based on the different modes of contact used. As defined in previous review, one in-person group or individual session was equivalent to 1 session, one online or telephone session was equivalent to 0.5 session and any contact via text, email, fax or newsletter was equivalent 0.25 session.(72)	To determine intervention characteristics associated with improved outcomes, an effectiveness ratio was calculated by dividing the number of interventions that were effective and used a particular intervention characteristic by the total number of interventions that used that characteristic for each criterion of the TIDieR checklist. These effectiveness ratios are presented as percentages with higher values indicating greater effectiveness of the characteristic. The effectiveness ratios were utilised for comparison of the efficacy of specific intervention characteristics when there were at least three studies featuring each intervention characteristic for each criterion of the TIDieR checklist to ensure reliable comparison.
Economic evaluation	To assess the economic evaluation of interventions, data was collected on whether each study conducted an economic evaluation of their interventions.	

Results on the efficacy of the interventions are presented narratively in two groups: studies that compared a behavioural intervention to a control group, and those that compared two behavioural interventions. Methods for data synthesis are described in Table 2.

## Results

### Description of included studies

**Fig. 1:**
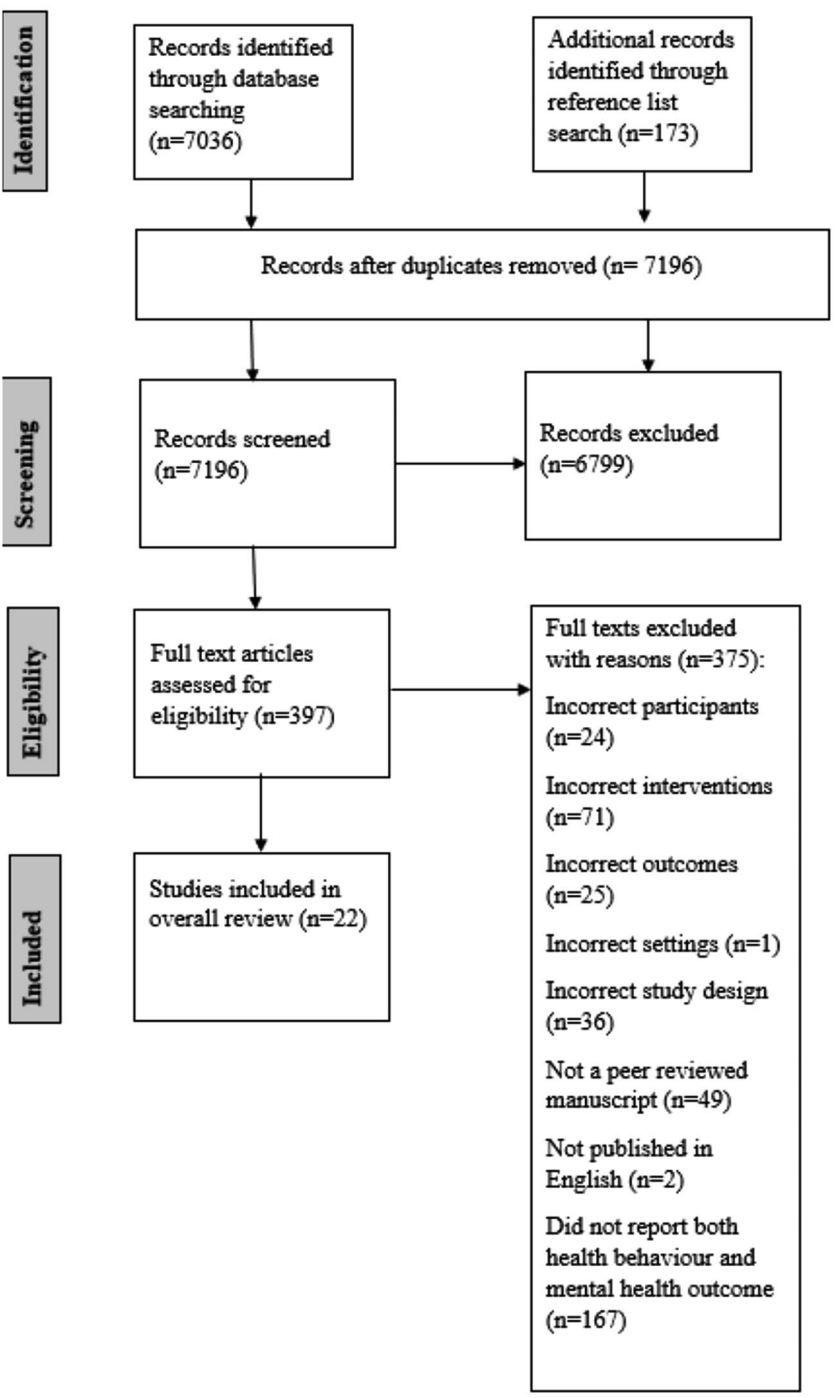
Flow diagram of included studies

A total of 7196 articles were identified (Fig. 1). After initial exclusion based on titles and abstracts, 397 full texts were screened for inclusion with, 22 studies included in this review.

A total of 11,044 participants (sample range: 34 to 3755) were included across 22 studies (Table 3). The mean age of participants was 20.5 (± 2.2) years. A higher proportion of studies (*n* = 10, 46%) did not restrict by age, recruiting all students over the age of 17. 95% of studies (*n* = 21) included both males and females, with mean of 65% of study participants being female. Seventeen studies (77%) had a health behaviour-related inclusion criteria for participants (e.g. participants were excluded if they had health conditions which restricted diet or physical activity or did not meet criteria for hazardous drinking/ insomnia) [32–48]. Sixteen studies (73%) had a student-related inclusion criteria (e.g. inclusion of current full time undergraduate students, exclusion of collegiate athletes or students majoring in nutrition, exercise science, and/or health promotion courses, students studying overseas) [33, 34, 36–40, 44–46, 48–53]. Only six studies (27%) had a mental health related inclusion criteria (e.g. exclusion if current or past medical history of mental disorder or psychiatric condition or current participation in mental health treatment) [37, 41, 42, 47, 49, 50]. Overall, 55% of studies were conducted in the United States (USA) (*n* = 12) and a majority were published between 2018 and 2023 (*n* = 13, 59%).

**Table 3:** Summary of study characteristics of 22 RCTs evaluating interventions targeting health behaviours in university students

		All studies
Country *n* (%)	United States	12 (54.5)
	China	3 (13.6)
	United Kingdom	2 (9.1)
	Australia	1 (4.5)
	Other	4 (18.2)
Publication Year *n* (%)	2012–2017	9 (40.9)
	2018–2023	13 (59.1)
Number of participants	Total	11,044
	Mean	502
	Median	139
	Range	34-3755
Age *n* (%)	Mean (SD)	20.5 (2.2)
	Range of mean age	18.0-26.8
	Number of studies that restricted by age: 16–24	5 (22.7)
	Number of studies that restricted by age: 17–35	4 (18.2)
	Number of studies that restricted by age: 20–42	1 (4.5)
	Number of studies unrestricted by age	10 (45.5)
	Number of studies that did not specify age range	2 (9.1)
Sex/ Gender	Mean Female (%)	65.2
	Range Female (%)	12.7–100.0
Participant: Health behaviour related inclusion criteria *n* (%)	Yes	17 (77.3)
	No	5 (22.7)
Participant: Student- related inclusion criteria *n* (%)	Yes	16 (72.7)
	No	6 (27.3)
Participant: Mental health related inclusion criteria *n* (%)	Yes	6 (27.3)
	No	16 (72.7)
Health Behaviour Outcome n(%)	Diet	9 (40.9)
	Physical Activity	10 (45.5)
	Sedentary Behaviour	0 (0.0)
	Sleep	9 (40.9)
	Alcohol Intake	7 (31.8)
	Smoking	1 (4.5)
	Drug Use	3 (13.6)
Mental Health Outcome *n* (%)	Depression	13 (59.1)
	Anxiety	9 (40.9)
	Stress	7 (31.8)
	Psychological Wellbeing	5 (22.7)
	General Wellbeing	2 (9.1)
	PTSD	1 (4.5)
Study Design *n* (%)	RCT	17 (77.3)
	Pilot RCT	5 (22.7)
Number of study arms *n* (%)	Two	20 (90.9)
	Three	2 (9.1)
Number of intervention arms *n* (%)	One	22 (84.6)
	Two	4 (18.2)
	Total	26
Type of control groups *n* (%)	No Intervention	9 (40.9)
	Standard/usual care	6 (27.3)
	Wait-list control	5 (22.7)
	No control group	2 (9.1)
Behavioural focus of intervention: Number of health behaviours of interest targeted per intervention arm *n* (%)	One	18 (69.2)
	Two	5 (19.2)
	Three	3 (11.5)
Behavioural focus of intervention: Type of behaviour targeted per intervention arm *n* (%)	Diet	10 (38.5)
	Physical Activity	9 (34.6)
	Sedentary Behaviour	0 (0.0)
	Sleep	10 (38.5)
	Alcohol Intake	7 (26.9)
	Smoking	0 (0.0)
	Drug Use	1 (3.8)
Intervention Duration *n* (%)	Brief	7 (26.9)
	≤ 5 weeks	3 (13.6)
	6 to ≤ 12 weeks	15 (57.7)
	Unclear	1 (4.5)

Across the 22 included RCTs, there were 26 different health behaviour interventions. Most studies (*n* = 18) included one intervention group compared to a control group [32, 34–38, 40, 42–47, 49–53]. Two studies [41, 48] compared two interventions while the remaining two studies [33, 39] compared two interventions to a control group. The number of health behaviours targeted ranged from one to three per intervention arm, with 70% of interventions targeting one health behaviour (*n* = 18, 69%) [32, 35, 37, 39–43, 45–52]. The most commonly targeted health behaviours were diet [33, 34, 36–38, 44, 48, 52, 53] (*n* = 10, 39%) and sleep [35, 41, 44, 47, 49–53] (*n* = 10, 39%), followed by physical activity [33, 34, 36, 38, 44, 45, 52, 53] (*n* = 9, 35%) and alcohol intake [39, 40, 42, 43, 46, 48] (*n* = 7, 27%). No interventions targeted smoking or sedentary behaviour. Seven studies [33, 34, 36, 38, 44, 52, 53] targeted multiple health behaviours with four interventions that targeted both diet and physical activity [33, 34, 36, 38] and three targeting diet, physical activity and sleep [44, 52, 53]. Intervention duration ranged from brief single session interventions to 12 weeks, with most running for 6 to 12 weeks. The number of data collection points ranged from two to five across all studies. Eight studies [32, 37, 41, 44–46, 52, 53] measured data pre- and post- intervention only while the remaining 14 studies [33–36, 38–40, 42, 43, 47–51] featured follow up at multiple timepoints post intervention. The most measured health behaviour outcomes were physical activity [33, 34, 36–38, 44, 45, 50, 52, 53] (*n* = 10, 46%), followed by diet [33, 34, 36–38, 44, 48, 50, 52] (*n* = 9, 41%) and sleep [35, 38, 41, 44, 47, 49–51, 53] (*n* = 9,41%) then alcohol [39, 40, 42, 43, 46, 48, 50] (*n* = 7, 32%). Depression [33–35, 37, 39–43, 46, 47, 49, 51] (*n* = 13, 59%), anxiety [32, 35, 37, 39, 41, 42, 46, 51, 53] (*n* = 9, 41%) and stress [36, 38, 39, 41, 44, 50, 51] (*n* = 7,32%) were the most common mental health outcomes measured across studies.

### Risk of bias assessment

**Fig. 2:**
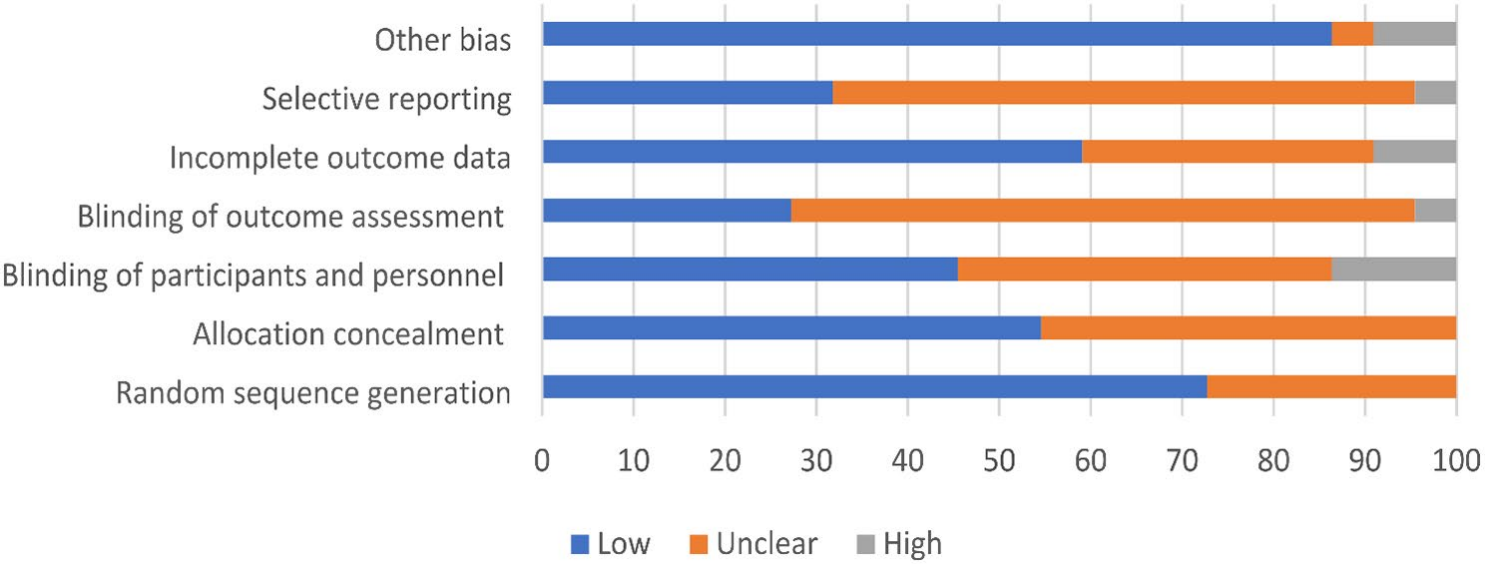
Risk of bias of included studies

Figure 2 summarises the risk of bias assessment of included studies. There was a low risk of bias for random sequence generation (*n* = 16,73%), allocation concealment (*n* = 12, 55%), incomplete outcome data (*n* = 13,59%) and source of other bias (n-19, 86%). However, most studies did not adequately describe blinding of outcome assessment (*n* = 15, 68%) and selective outcome reporting (*n* = 14, 64%). In terms of blinding of participants and personnel, while most studies did feature a low risk of bias (*n* = 10,45%), a significant amount did not adequately describe blinding methods (*n* = 9, 41%).

### Efficacy of interventions in improving health behaviour and mental health outcomes

All statistically significant findings in this review were in the desired direction (e.g. improved dietary intake, reduced alcohol consumption etc.). A summary of outcomes across all studies can be found in Table 4.

**Table 4:** Summary of outcomes of 22 RCTs targeting health behaviours

Study	Intervention	Data Collection Timepoints (weeks)	Health Behaviour Outcome							Mental Health Outcome					
			Diet	*P*.A	S.B	Sleep	Alcohol Intake	Smoking	Drug Use	Depression	Anxiety	Stress	Psychological Wellbeing	General Wellbeing	PTSD
Intervention vs. Control															
Hahn, 2021	Diet	0,4	NS	NS	x	x	x	x	x	NS	NS	x	x	x	x
Sharp, 2016	Exercise	0,12	x	NS	x	x	x	x	x	x	x	x	NS	x	x
Taylor, 2014	Sleep	0, 6, 13	x	x	x	**S- 6wks only**	x	x	NS	NS	x	x	x	x	x
Freeman, 2017	Sleep	0,3,10,22	x	x	x	**S**	x	x	x	**S**	**S**	x	**S**	x	x
Hershner, 2018	Sleep	0, 1, 8	x	x	x	**S- 8wks only**	x	x	x	**S-8 weeks only**	x	x	x	x	x
Huberty, 2019	Sleep	0,8,12	NS	NS	x	NS	NS	x	x	x	x	**S**	x	x	x
Spanhel, 2022	Sleep	0,4,12	x	x	x	**S**	x	x	x	**S- 12 weeks only**	NS	NS	x	x	x
Murphy, 2012	Alcohol Intake	0,4, 20 or 30	x	x	x	x	NS	x	x	NS	x	x	x	x	x
Pengpid, 2013	Alcohol Intake	0,26,48	x	x	x	x	**S**	NS	NS	NS	x	x	x	x	NS
Murphy, 2019	Alcohol Intake	0, 4, 26, 52, 69	x	x	x	x	**S**	x	x	**S**	**S**	NS	x	x	x
Murphy, 2019	Alcohol Intake	0, 4, 26, 52, 69	x	x	x	x	**S**	x	x	**S**	**S**	NS	x	x	x
Paulus, 2021	Alcohol Intake	0,1,4,13	x	x	x	x	**S- 13 weeks only**	x	x	NS	NS	x	x	x	x
Shuai, 2022	Alcohol Intake	0,2	x	x	x	x	NS	x	x	**S**	NS	x	x	x	x
Buckner, 2020	Drug Use	0,2	x	x	x	x	x	x	**S**	x	**S**	x	**S**	x	x
Greene, 2012	Diet and Exercise	0, 13, 65	**S**	**S**	x	x	x	x	x	x	x	**S**	**S**	x	x
Kattelman,2014	Diet and Exercise	0, 13, 64	**S**	NS	x	**S**	x	x	x	x	x	NS	x	x	x
Duan, 2017	Diet and Exercise	0, 8, 12	**S**	NS	x	x	x	x	x	NS	x	x	x	x	x
Duan, 2022	Diet and Exercise	0,4,8,12	**S**	**S**	x	x	x	x	x	NS	x	x	x	x	x
Duan, 2022	Diet and Exercise	0,4,8,12	**S**	**S**	x	x	x	x	x	NS	x	x	x	x	x
Sandrick, 2017	Diet, Exercise, Sleep	0,8	NS	**S**	x	NS	x	x	x	x	x	NS	x	x	x
Yang, 2020	Diet, Exercise, Sleep	0,30	**S**	**S**	x	x	x	x	x	x	x	x	x	NS	x
Yan, 2023	Diet, Exercise, Sleep	0,8	x	**S**	x	NS	x	x	x	x	NS	x	NS	x	x
Intervention vs. Intervention															
Okajima, 2022	Sleep	0,8	x	x	x	**S**	x	x	x	**S**	**S**	NS	x	x	x
Duan, 2022	Diet and Exercise	0,4,8,12	**S**	NS	x	x	x	x	x	NS	x	x	x	x	x
Whatnall, 2019	Diet vs. Alcohol Intake	0,13	**S**	x	x	x	NS	x	x	x	x	x	x	NS	x
Murphy, 2019	Alcohol Intake	0, 4, 26, 52, 69	x	x	x	x	NS	x	x	NS	NS	NS	x	x	x

All significant outcomes were in the hypothesized direction and have been highlighted in bold text. P.A – physical activity; S.B – sedentary behaviour; PTSD – post traumatic stress disorder; S: significant; NS: not significant; x: outcome not assessed

#### Health behaviour interventions vs. control

Twenty studies [32–40, 42–47, 49–52] evaluated a health behaviour intervention compared with a control group (no intervention, standard/usual care, waitlist control group or no control), with six studies [32, 35, 36, 39, 49, 51] (30%) finding significant improvements in both health behaviour and mental health outcomes.

Five studies [35, 47, 49–51] (25%) evaluated sleep interventions, of which three [35, 49, 51] reported significant improvements in both sleep outcomes (sleepiness, sleep quality and insomnia severity) and mental health outcomes (anxiety, depressive symptoms and psychological wellbeing) in the intervention group when compared with the control group. These interventions ranged from 3 to 10 weeks in duration, with two studies featuring online cognitive behavioural therapy interventions and one featuring an online sleep education intervention. Of the two remaining studies, one [47] found significant improvements in sleep outcomes including sleep efficiency, daytime functioning and insomnia severity from pre-treatment to post treatment while the other study [50] only found significant improvements in mental health outcomes including perceived stress across all timepoints.

Five RCTs [39, 40, 42, 43, 46] (25%) evaluated alcohol interventions, of which only one [39] found significant improvements in alcohol consumption as well as anxiety and depressive symptoms across four timepoints (i.e. 1, 6, 12 and 16 months) [39]. Murphy et al. evaluated two alcohol interventions; a brief motivational intervention supplemented with substance-free activity sessions and a brief motivational intervention supplemented with relaxation training compared to a control group. Of the remaining studies, two [42, 43] found significant improvement in alcohol consumption, heavy episodic drinking, and hazardous alcohol use, but not in mental health outcomes, while one study [46] found significant improvements only in depressive symptoms.

One RCT (5%) evaluated a brief online personalized feedback intervention aiming to decrease cannabis use which found significant improvements in both cannabis use frequency and mental health outcomes including social anxiety as well as positive and negative affect when intervention group was compared with control group [32].

Two RCTs (10%) targeted diet and physical activity separately and did not find significant improvements in either health behaviour outcomes or mental health outcomes [37, 45]. One study featured a 4-week dietary self-monitoring intervention while the other featured a 12-week pedometer-based intervention.

Four RCTs (20%) evaluated interventions targeting nutrition and physical activity behaviour change [33, 34, 36, 38]. Only one study which evaluated a 10-week online education intervention with curriculum focused on improving attitudes, behaviours and self-efficacy in facilitating weight management found statistically significant improvements in fruit and vegetable consumption, amount of physical activity performed per week as well as improvements in emotional problems and stress across all timepoints) [36]. All three of the remaining studies found significant improvements in health behaviour outcomes only including fruit and vegetable consumption, fat consumption, amount of physical activity performed and number of hours of sleep [33, 34, 38].

Three RCTs (15%) evaluated interventions targeting nutrition, physical activity, and sleep behaviour change of which none found a significant improvement in health behaviours and mental health outcomes [44, 52, 53]. Only one study evaluating a 7-week health education intervention found a significant improvement in nutrition outcomes, including consumption of breakfast and sugar-sweetened beverages between groups [52]. All studies found significant improvement in amount of physical activity performed between groups across all timepoints [44, 52]. The interventions included 7-week health education intervention, an 8 week health counselling intervention supplemented by health messages delivered via text message and an 8-week peer health coaching intervention. Two studies assessed sleep outcomes which found no significant changes between groups [44, 53]. In terms of mental health outcomes, one study [44] assessed stress, one [52] assessed general wellbeing and the other [53] assessed depression and psychological wellbeing with none finding significant changes in outcomes between groups.

#### Health behaviour intervention vs. health behaviour intervention

Four RCTs [33, 39, 41, 48] (18%) compared the efficacy of two health behaviour interventions in improving health behaviour and mental health outcomes of which only two [39, 41] found significant improvements in both health behaviour and mental health outcomes. One RCT (25%) evaluated two sleep interventions; a cognitive-behavioural therapy (CBT) intervention for insomnia compared to a sleep self-monitoring intervention [41]. The study found significant improvements in sleep outcomes including insomnia severity, sleep hygiene practices, pre-sleep arousal as well as mental health outcomes including depression and anxiety in the CBT group compared to the self-monitoring group post intervention [41]. Murphy et al. evaluated two alcohol interventions as specified above, however no difference in alcohol or mental health outcomes were observed between intervention groups [39].

One RCT (25%) compared nutrition and physical activity intervention whereby one group received the nutrition module first then the physical activity module and the other group the alternate [33]. The study found a significant difference in nutrition outcomes with the group receiving the nutrition module first having higher fruit and vegetable consumption compared with the other group at final follow up [33]. Both interventions found significant improvements in amount of physical activity performed compared with control but there were no significant differences between intervention groups. The study also assessed depression and depressive symptoms, but no significant changes were found between intervention groups [33].

One RCT (25%) compared a diet intervention with an alcohol intervention. Both interventions were brief web-based interventions with the diet intervention being designed based on the social cognitive theory and the theory of planned behaviour [48]. A significant decrease in percentage energy from discretionary foods was observed in the diet intervention group compared to the alcohol intervention group post intervention [48]. There were no significant differences in alcohol outcomes or mental health outcomes between groups.

### Intervention impact (i.e., penetration, fidelity, and implementation)

Table 5 summarises intervention impact using the PIPE Impact Metric. Only three studies provided sufficient information to approximate penetration rate which ranged from 18 to 53% [34, 37, 49]. The majority of included interventions featured low program fidelity [32–38, 41, 42, 44–46, 48–53] (*n* = 18), one study [43] featuring moderate program fidelity and three studies [39, 40, 47] featuring high program fidelity. For participation, eighteen studies provided sufficient information, with participation rates ranging from 7 to 89% [33–44, 46–51, 53]. For effect, as previously reported, seven studies found a significant improvement in both health behaviour change and mental health related outcome [32, 35, 36, 39, 41, 49, 51].

**Table 5:** Penetration, participation, implementation, and effectiveness of randomized controlled health behaviour change trials in university students

Study	Country	Age Range	Sample Size (baseline)	Intervention	Penetration Rate (%)	Implementation (Fidelity)	Participation Rate (%)	Effectiveness	
								Health Behaviour	Mental Health
Intervention vs. Control									
Hahn, 2021	US	≥ 18	200	Diet	18	Low	25	NS	NS
Sharp,2016	Canada	≥ 17	184	Exercise	NAC	Low	NAC	NS	NS
Taylor, 2014	US	18–27	34	Sleep	NAC	High	20	S	NS
Freeman,2017	UK	≥ 18	3755	Sleep	NAC	Low	44	S	S
Hershner,2018	US	≥ 18	549	Sleep	28	Low	81	S	S
Huberty, 2019	US	≥ 18	109	Sleep	NAC	Low	33	NS	S
Spanhel, 2022	Germany	20–42	81	Sleep	NAC	Low	59	S	S – depression only
Murphy,2012	US	18–21	82	Alcohol Intake	NAC	High	7	NS	NS
Pengpid,2013	South Africa	≥ 18	152	Alcohol Intake	NAC	Moderate	21	S- alcohol intake only	NS
Murphy,2019	US	N	393	Alcohol Intake	NAC	High	7	S	S – depression and anxiety only
Paulus, 2021	US	≥ 18	125	Alcohol Intake	NAC	Low	12	S	NS
Shuai,2022	UK	18–25	76	Alcohol Intake	NAC	Low	34	NS	S – depression only
Buckner,2020	US	≥ 18	102	Drug Use	NAC	Low	NAC	S	S
Greene,2012	US	18–24	1689	Diet and Exercise	NAC	Low	19	S	S
Kattelman,2014	US	18–24	1639	Diet and Exercise	NAC	Low	26	S- diet and sleep only	NS
Duan, 2017	China	17–24	493	Diet and Exercise	53	Low	87	S- diet only	NS
Duan,2022	China	≥ 18	565	Diet and Exercise	NAC	Low	89	S	NS
Sandrick, 2017	US	18–30	60	Diet, Exercise, Sleep	NAC	Low	71	S – exercise only	NS
Yang,2020	China	16–24	532	Diet, Exercise, Sleep	NAC	Low	NAC	S	NS
Yan,2023	US	≥ 18	52	Diet, Exercise, Sleep	NAC	Low	NAC	S-exercise only	NS
Intervention vs. Intervention									
Okajima, 2022	Japan	N	48	Sleep	NAC	Low	27	S	S- depression and anxiety only
Duan,2022	China	≥ 18	565	Diet and Exercise	NAC	Low	89	S- diet only	NS
Whatnall,2019	Australia	17–35	124	Diet and Alcohol Intake	NAC	Low	41	S-diet only	NS
Murphy,2019	US	N	393	Alcohol Intake	NAC	High	7	NS	NS

Low fidelity: no manual or checklist; Moderate fidelity: only manual but no checklist or other measures of quality assurance; NAC: not able to calculate; NS: not significant; S: significant improvements compared to control

### Intervention characteristics associated with improved outcomes (efficacy)

Intervention characteristics as per the TIDieR checklist are shown in Table 6.

**Table 6:** Intervention characteristics of health behaviour interventions in university students as per TIDieR checklist abbreviations: RR, retention rate; BMI, brief motivational interviewing; SFAS, substance free activity session; MI: motivational interviewing

Study; Sample Size	Participant Age (range)	Intervention type	Theory	Who; Where	How	When; How Much	Tailoring	Modification	How Well
Intervention vs. Control									
Hahn, 2021 *N* = 200	≥ 18	Diet	Dietary Self-Monitoring	Trained research staff, Combination	Individual, In person + Technology	4 weeks, 9.5 sessions	No	No	RR post intervention: 96.0% RR final follow up: NA Fidelity– Planned: Low Actual: NA
Sharp,2016 *N* = 184	≥ 17	Exercise	NA	Not specified, Researcher-based	Individual, In person + Technology	12 weeks, 2 sessions	No	No	RR post intervention: 74.5% RR final follow up: NA Fidelity– Planned: Low Actual: NA
Taylor, 2014 *N* = 34	18–27	Sleep	Cognitive Behavioural Therapy (CBT)	Graduate Student, Researched-based	Individual, In person	6 weeks, 6 sessions	No	No	RR post intervention: 85.3% RR final follow up: 20.6% Fidelity– Planned: High– The evaluators rated each component specified in the treatment manual in each session on a 5-point Likert -type scale (0 = poor/absent, 2 = present/acceptable,4 = excellent). Actual: Mean rating for adherence of evaluated sessions was 2.85 (SD – 0.56). Overall mean of 90.49% (SD- 14.78) of required components present per treatment manual.
Freeman,2017 *N* = 3755	≥ 18	Sleep	Cognitive Behavioural Therapy (CBT)	Automated Delivery, Participant-based	Individual, Technology	10 weeks, 3 sessions	Yes	No	RR post intervention: 48.6% RR final follow up: 41.9% Fidelity– Planned: Low Actual: NA
Hershner,2018 *N* = 549	≥ 18	Sleep	NA	Automated Delivery, Participant-based	Individual, Technology	8 weeks, Unclear	Yes	No	RR post intervention: 63.9% RR final follow up: 65.2% Fidelity– Planned: Low Actual: NA
Huberty, 2019 *N* = 109	≥ 18	Sleep	Mindfulness Based Stress Reduction (MBSR), Mindfulness-Based Cognitive Therapy (MBCT)	Automated Delivery, Participant-based	Individual, Technology	8 weeks, 28 sessions	No	No	RR post intervention: 83.5% RR final follow up: 55.0% Fidelity– Planned: Low Actual: NA
Spanhel, 2022 *N* = 81	20–42	Sleep	NA	Automated Delivery, Participant-based	Individual, Technology	3 weeks, 1.5 sessions	Yes	No	RR post intervention: NA RR final follow up: 64.2% Fidelity– Planned: Low Actual: NA
Murphy,2012 *N* = 82	18–21	Alcohol Intake	Behavioural Motivation & Behavioural Economic Supplement	Graduate student, Researched-based	Individual, In person	Unclear, 2 sessions	Yes	No	RR post intervention: 100% RR final follow up: 86.6% Fidelity– Planned: High Each of the components on the protocol was rated as a 1 “Did it poorly or didn’t do it but should have,” 2 “Meets Expectations,” or 3 “Above Expectations”. A score of 2 or higher indicated that the intervention component was delivered in a manner that was consistent. with the protocol in terms of both content and motivational interviewing style. Actual: BMI protocol adherence: Mean rating 1.89 (SD- 0.35). 92% of components meeting/ exceeding expectations MI skills in BMI adherence: Mean rating 2.00 (SD- 0.19). 93% of components meeting / exceeding expectations SFAS protocol adherence: Mean rating 1.91 (SD- 0.31). 91% of components meeting / exceeding expectations MI skills in SFAS adherence: Mean rating 1.96 (SD- 0.17). 90% of components meeting / exceeding expectations Relaxation session adherence: Mean rating 2.03 (SD- 0.07). 93% of components meeting / exceeding expectations
Pengpid,2013 *N* = 152	≥ 18	Alcohol Intake	Information-Motivation-Behavioural Skills (IMB) Model	Research assistant nurses, Researcher-based	Individual, In person	Brief intervention, 1 session	Yes	No	RR post intervention: NA RR final follow up: 96.7% Fidelity– Planned: Moderate Actual: At least 13/15 requisite intervention steps were implemented in 82% of intervention sessions
Paulus, 2021 *N* = 125	≥ 18	Alcohol Intake	Personalised Feedback	Automated Delivery, Participant-based	Individual, Technology	Brief intervention, 0.5 sessions	Yes	No	RR post intervention: 84.0% RR final follow up: 67.2% Fidelity– Planned: Low Actual: NA
Shuai,2022 *N* = 76	18–25	Alcohol Intake	Functional Imagery Training (FIT)	Automated Delivery, Participant-based	Individual, Technology	2 weeks, 4 sessions	No	No	RR post intervention: 68.4% RR final follow up: NA Fidelity– Planned: Low Actual: NA
Buckner,2020 *N* = 102	≥ 18	Drug Use	Personalised Feedback	Automated Delivery, Participant-based	Individual, Technology	Brief intervention, 0.5 sessions	Yes	No	RR post intervention: 61.8% RR final follow up: NA Fidelity– Planned: Low Actual: NA
Greene,2012 *N* = 1689	18–24	Diet and Exercise	Dick and Carey’s System of Instructional Design and Keller’s Instructional Motivational Model	Automated Delivery, Participant-based	Individual, Technology	10 weeks, 5 sessions	No	No	RR post intervention: 79.8% RR final follow up: 66.7% Fidelity– Planned: Low Actual: NA
Kattelman,2014 *N* = 1639	18–24	Diet and Exercise	PRECEDE-PROCEED & Dick and Carey’s Model of Instructional Design	Automated Delivery, Participant-based	Individual, Technology	10 weeks, 20.5 sessions	Yes	No	RR post intervention: 75.7% RR final follow up: 59.4% Fidelity– Planned: Low Actual: NA
Duan, 2017 *N* = 493	17–24	Diet and Exercise	Health Action Process Approach (HAPA)	Automated Delivery, Participant-based	Individual, Technology	8 weeks, 4 sessions	Yes	No	RR post intervention: 68.4% RR final follow up: 28.8% Fidelity– Planned: Low Actual: NA
Sandrick, 2017 *N* = 60	18–30	Diet, Exercise, Sleep	NA	Health professional, Combination	Individual, In person + Technology	8 weeks, 9 sessions	Yes	No	RR post intervention: 100.0% RR final follow up: 100.0% Fidelity– Planned: Low Actual: NA
Yang,2020 *N* = 532	16–24	Diet, Exercise, Sleep	Social Cognitive Theory	Instructor, Researcher-based	Group, In person	7 weeks, 7 sessions	No	No	RR post intervention: NA RR final follow up: NA Fidelity– Planned: Low Actual: NA
Yan, 2023 *N* = 52	≥ 18	Diet, Exercise, Sleep	NA	Peer, Researcher-based	Individual, In person	8 weeks, 8 sessions	Yes	No	RR post intervention: 82.7 RR final follow up: NA Fidelity– Planned: Low Actual: NA
Intervention vs. Intervention									
Okajima, 2022 *N* = 48	N	Sleep	Cognitive Behavioural Therapy (CBT)	Automated Delivery, Participant-based	Individual, Technology	8 weeks, 4 sessions	No	No	RR post intervention: 85.4% RR final follow up: NA Fidelity– Planned: Low Actual: NA
		Sleep	NA	Automated Delivery, Participant-based	Individual, Technology	8 weeks, 2 sessions	No	No	
Duan,2022 *N* = 565	≥ 18	Diet and Exercise	Health Action Process Approach (HAPA)	Not specified, Researcher-based	Individual, In person	8 weeks, 8 sessions	No	No	RR post intervention: 74.3% RR final follow up: 63.0% Fidelity– Planned: Low Actual: NA
		Diet and Exercise	Health Action Process Approach (HAPA)	Not specified, Researcher-based	Individual, In person	8 weeks, 8 sessions	No	No	
Whatnall,2019 *N* = 124	17–35	Diet	PRECEDE-PROCEED, Social Cognitive Theory, Theory of Planned Behaviour	Automated Delivery, Participant-based	Individual, Technology	Brief intervention, 0.25 sessions	Yes	No	RR post intervention: 72.6% RR final follow up: NA Fidelity– Planned: Low Actual: NA
		Alcohol Intake	Personalized Feedback	Automated Delivery, Participant-based	Individual, Technology	Brief intervention, 0.25 sessions	Yes	No	
Murphy,2019 *N* = 393	N	Alcohol Intake	Behavioural Motivation & Behavioural Economic Supplement	Graduate student, Researcher-based	Individual, In person	Brief intervention, 2 sessions	Yes	No	RR post intervention: 93.1% RR final follow up: 79.1% Fidelity– Planned: High Each of the components on the protocol was rated as a 1 “Did it poorly or didn’t do it but should have,” 2 “Meets expectations,” or 3 “Above Expectations” Actual: BMI protocol adherence: Mean rating 1.94 (SD- 0.23). 88% of components meeting/ exceeding expectations SFAS protocol adherence: Mean rating 1.85 (SD- 0.42). 87% of components meeting / exceeding expectations Relaxation session adherence: Mean rating 2.27 (SD- 0.47). 99% of components meeting / exceeding expectations MI treatment integrity: All codes demonstrated acceptable reliability.
		Alcohol Intake	Behavioural Motivation & Behavioural Economic Supplement	Graduate student, Researcher-based	Individual, In person	Brief intervention, 2 sessions	Yes	No	

#### Why (theoretical framework)

Overall, a higher proportion of interventions (*n* = 20, 77%) specified a theory used to underpin the intervention [32–43, 46–48, 50]. Interventions that did not report the utilisation of a theoretical framework [41, 45, 49, 51–53] had an effectiveness ratio of 50%, whereas those that did had an effectiveness ratio of 30%.

#### Who (intervention provider)

Most interventions were delivered via automated delivery (*n* = 14, 54%) such as email or a website [32, 34–36, 38, 41, 42, 46, 48–51]. Other intervention providers included trained research staff [37] (*n* = 1, 4%), graduate students [39, 40, 47] (*n* = 4, 15%), research assistant nurses [43] (*n* = 1, 4%), health professionals [44] (*n* = 1, 4%), peers (*n* = 1,4%) and instructors [52] (*n* = 1,4%). Interventions that were delivered via automated delivery or graduate students had an effectiveness ratio of 50%, while the effectiveness ratio could not be calculated for the other intervention providers due to the low number of studies.

#### Where (intervention location)

Fourteen interventions [32, 34–36, 38, 41, 42, 46, 48–51] (54%) were conducted in a participant-based environment as they were delivered online while ten interventions [33, 39, 40, 43, 45, 47, 52, 53] (38%) were conducted in researcher- based environments. Two studies (8%) used a combined approach [37, 44]. Interventions that utilized a participant-based location had an effectiveness ratio of 50% while interventions delivered in researcher-based environments had an effectiveness ratio of 20%.

#### How (delivery format and mode of delivery)

Twenty-five interventions [32–51, 53] (96%) were delivered in an individual format and only one was delivered in a group setting [52]. Interventions delivered in an individual format had an effectiveness ratio of 36%. Fourteen interventions [32, 34–36, 38, 41, 42, 46, 48–51] (54%) were delivered using technology, nine [33, 39, 40, 43, 47, 52, 53] (35%) were delivered in person and three [37, 44, 45] (12%) utilized a combination of both in intervention delivery. Interventions delivered using technology and in person had an effectiveness ratio of 50% and 22% respectively.

#### When and how much (duration and number of sessions)

Intervention duration ranged from brief single session interventions to 12 weeks. Brief interventions [32, 39, 42, 43, 48] (*n* = 7, 27%) had an effectiveness ratio of 43% while those that ran 2 to 5 weeks [37, 46, 51] (*n* = 3, 12%) had an effectiveness ratio of 33% and those that ran for up to 12 weeks [33–36, 38, 41, 44, 45, 47, 49, 50, 52, 53] (*n* = 15, 58%) had an effectiveness ratio of 33%. The number of sessions used to deliver interventions ranged from 0.25 to 28 sessions. Interventions [32, 34, 35, 39–43, 45, 46, 48, 51] (*n* = 15, 58%) that were delivered in less than five sessions had an effectiveness ratio of 47% while those delivered in 5–10 sessions [33, 36, 37, 44, 47, 52, 53] (*n* = 8, 31%) had a ratio of 13%.

#### Tailoring and modification

Most studies [32, 34, 35, 38–40, 42–44, 48, 49, 51, 53] (*n* = 15,58%) tailored interventions to provide individualized goal setting or personalized feedback for participants on engagement in the targeted health behaviour(s). Tailored interventions [32, 34, 35, 38–40, 42–44, 48, 49, 51, 53] had an effectiveness ratio of 40% while non-tailored interventions [33, 36, 37, 41, 45–47, 50, 52] had a ratio of 27%.None of the included studies reported modifying interventions throughout implementation.

#### How well (retention rate and fidelity)

Retention rate post intervention ranged from 49 to 100% with a mean retention rate of 79% (*n* = 19, 73%). Retention rates at final follow up ranged from 21 to 100% with a mean retention rate of 64% (*n* = 16, 62%). Studies [32–34, 36, 38, 45, 46, 48, 49] (*n* = 9, 35%) with retention rate between 50 and 80% post intervention had an effectiveness ratio of 33% while studies [37, 39–42, 44, 47, 50] (*n* = 8, 31%) with a retention rate of more than 80% post intervention had an effectiveness ratio of 22%. None of the studies had a retention rate of less than 50% post intervention. Studies [33, 36, 38, 39, 42, 49–51] (*n* = 8,31%) with a retention rate between 50 and 80% at final follow up had an effectiveness ratio of 40% compared to 33% for studies [34, 35, 47] (*n* = 3, 12%) that had a retention rate of less than 50% and greater than 80% at final follow up. Most studies (*n* = 18, 69%) reported low fidelity [32–38, 41, 42, 44–46, 48–53]. The few studies that assessed intervention compliance reviewed session recordings to assess adherence to defined protocol. Both studies with low [32–38, 41, 42, 44–46, 48–53] and high [39, 40, 47] fidelity had an effectiveness ratio of 33% respectively. Fidelity measures and results are described in Table 6.

### Economic evaluation

None of the included studies conducted an economic evaluation.

## Discussion

This systematic review of 22 RCTs is the first to synthesize evidence on the efficacy, impact, and economic evaluation of health behaviour interventions to improve both health behaviour and mental health outcomes among students in the university setting. Only one third (*n* = 7) of studies were effective in improving both health behaviour and mental health outcomes, with most (*n* = 4) focused on improving sleep behaviours. Other effective interventions targeted diet and physical activity (*n* = 1), alcohol use (*n* = 1) and substance use (*n* = 1) behaviours. Due to inadequate reporting of outcomes, insufficient evidence was found regarding intervention impact, intervention characteristics associated with improved outcomes and the economic evaluation of interventions to guide the implementation of health behaviour interventions in the university setting. This review highlights the limited evidence base supporting the efficacy of health behaviour interventions in improving both health behaviour and mental health outcomes. Given the bidirectional link between mental health and health risk behaviours, effective interventions should consider holistic and integrated approaches to address engagement in health risk behaviours and mental health outcomes. Most of the included RCTs targeted similar health behaviours while none of the included studies aimed to improve smoking rates or sedentary behaviour. Most RCTs featured single behaviour interventions which predominantly targeted alcohol intake (*n* = 5) and sleep (*n* = 5). Whereas health behaviours such as diet and physical activity were predominantly targeted together in multi-behaviour interventions. Therefore, the review provides limited to no evidence demonstrating the efficacy of interventions targeting a range of single and multi-health behaviour interventions. Future research should focus on examining the efficacy of a range of health behaviour interventions with a specific focus on nutrition, sedentary behaviour, smoking, and substance use behaviour change, for which the evidence is currently limited as well as exploring different combinations of health behaviour interventions to gain an understanding of which health behaviours are most effective in improving both health behaviour and mental health outcomes.

Of note, sleep interventions exhibit potential in improving both sleep behaviours and mental health outcomes among university students, with four out of five studies proving effective. These findings parallel those of previous systematic reviews and meta-analyses which demonstrate the role of sleep in the onset and aggravation of various mental illnesses and the benefits of cognitive behavioural therapy interventions (CBT-I) as a non-pharmacological approach to improve sleep and mental health [11, 54, 55]. The included studies targeted a range of sleep variables including sleep quality, sleep disturbances (e.g. insomnia severity, daytime functioning, pre-sleep arousal), sleep efficiency and sleep hygiene practices. Studies that were effective found significant changes in depressive symptoms, anxiety, and psychological wellbeing.

Given the well- documented impacts of smoking and sedentary behaviour on mental and physical health, the lack of interventions addressing these behaviours in university students is surprising. In Australia, approximately 80% of long-term adult smokers begin smoking before the age of 20 due to stress or peer pressure, highlighting the need to address stress coping strategies in young adults [56]. Sedentary behaviour is now acknowledged as an independent factor from physical activity. Sedentary time negatively influences mental health by increasing the risk of anxiety, depression, and lowering levels of emotional wellbeing in diverse populations including younger adults [57, 58]. Thus, it is crucial for these behaviours to be addressed with equal importance in future interventions to ensure student mental health is being considered from all aspects of health and wellbeing.

There is strong evidence of co-occurrence of health risk behaviours among university students and emerging evidence suggesting an association between co-occurrence and mental ill health. As previously noted, only seven RCTs evaluated multiple health behaviour interventions. We observed that one out of seven (14%) multi-behaviour interventions were effective in improving both health behaviour and mental health outcomes compared to six out of fifteen (40%) single behaviour interventions. This suggests that there may be a higher likelihood in improving both outcomes when the intervention focuses on a single health behaviour. These findings contradict existing literature which infer that multi-behaviour interventions are potentially more effective due to the clustering of health risk behaviours [59–61]. However, the limited number of multi-behaviour interventions included in the review, and no studies that compared single behaviour to multiple behaviour interventions, hinders our ability to draw definitive conclusions. Therefore, future studies should directly compare a single behaviour approach to a multi-behaviour approach.

This systematic review showed that penetration, implementation, and participation are rarely addressed in RCTs evaluating health behaviour interventions targeting university students. Only three studies reported data to determine penetration rate. Considering penetration prior to implementation is central in designing health behaviour interventions for young adults, to ensure optimised utilisation of time and resources. Most studies reported the number of individuals reached by an invitation to participate in the study, many did not report the size of the overall target group. Thus, the penetration rate of health behaviour interventions in the university setting remains unknown. A recent review indicated that one- third of young adults who express interest and/or are screened for eligibility then provide consent and participate in the study [62]. However in this review, most studies (*n* = 18) reported data to determine participation rates, with a large variation of 7–89% observed in participation rate across studies. Measuring intervention fidelity or implementation enables greater transparency in assessing intervention effect and how quality standards were maintained. Thus, incorporating fidelity measures within RCTs equips the intervention for scale-up and can inform potential reasons for any loss of effect during implementation. 80% of studies reported low implementation fidelity measures with most not conducting a fidelity assessment of interventions. The assessment of implementation outcomes, such as penetration, participation and fidelity are central to assessing implementation success and processes. A recent study by Lengnick-Hall et al. reviewed 358 empirical studies to identify current reporting challenges of implementation outcomes and provided practical recommendations to address these [63]. To improve result validity and guide the implementation of health behaviour interventions in the university setting, future research should focus on: (1) defining and applying definitions of implementation outcome concepts explicitly throughout manuscript sections. (2) specifying analysis strategies for each implementation outcome relative to other constructs (3) identifying the reference point for measuring each implementation outcome (4) reporting the data provider, the level at which data will be collected and type of data to be collected for each implementation outcome (5) describing the number of timepoints and frequency of outcome measurement (6) specifying the unit of observation and unit of analysis for each implementation outcome [63].

There was some consistency across the included studies in terms of intervention characteristics. Most interventions were found to utilise a theory-based approach (*n* = 20), be delivered via automated means (*n* = 14), use a participant- based location (*n* = 14), provide tailored or personalised interventions (*n* = 21) and feature an individual delivery format (*n* = 24) as opposed to groups. There is no comprehensive data to support the use of a sole modality to improve both health behaviour and mental health outcomes among university students, or the general population. The use of consistent approaches across studies could be attributed to the familiarity of young adults in accessing information via technology and the cost effectiveness of many of these intervention characteristics. While only one third of interventions were deemed effective overall, a higher effectiveness ratio was observed for certain intervention characteristics, specifically interventions that did not utilise a theoretical framework (ER: 60%), were delivered via automated means (ER:50%), utilised a participant-based approach (ER:70%), were brief interventions (ER:43%), and were tailored to participants (ER:43%). Our findings indicate that despite the suitability of the approaches, not all intervention characteristics may be contributing to increasing the efficacy of interventions. For example, the efficacy of interventions that did not utilize a theoretical framework may be reflective of limitations in this systematic review’s methodology. Specifically, the review extracted data to indicate whether interventions utilized a theoretical framework, and the type of theory used. However, it did not explore the effects of different theoretical frameworks in relation to intervention efficacy within data analysis. Thus, future research should focus on exploring specific intervention characteristics associated with improved health behaviour and mental health outcomes in this target group/setting.

No studies included within this review reported an economic evaluation. Understanding the cost effectiveness of health behaviour interventions is imperative to maximise investments to facilitate equitable access to higher education and support retention and completion outcomes for this at-risk population group [2, 4]. While widespread in the clinical setting, limited evidence exists on the cost-effectiveness of implementing public health interventions [64]. A systematic review of economic evaluations applied to public health interventions indicated that over a 27-year period (1990–2017), only 14 studies reported on cost effectiveness [64]. This paucity of economic evaluations may be attributed to four methodological challenges including difficulties in attributing effects, measuring and valuing long-term outcomes, identifying intersectoral costs and consequences, and considering population health inequalities [64, 65]. These factors are compounded by a lack of standardized methodologies evaluating cost effectiveness and limited resources, perpetuating the underutilization of economic evaluations in this research space [66]. To address deficiencies in the application of economic evaluation methods, Reeves et al. has developed a short checklist to provide practical guidance for conducting and reporting on economic evaluations of public health intervention implementation [64].

### Strengths and limitations of included studies

The risk of bias assessment identified several strengths and limitations within included studies.

Generally, studies provided sufficient detail on randomization techniques, adequate generation of allocation sequence and management of study attrition. However, only half of the studies featured low risk of bias with many studies lacking sufficient detail regarding blinding of participants, personnel and/or outcome assessor and selective outcome reporting which limited our ability to determine study quality for most studies. The measurement methods used for most outcomes across studies were self-report with most utilising validated tools. Thus, while potential bias from self-report outcomes may have influenced the results of individual studies the use of validated self-report tools does strengthen the validity of findings. Furthermore, there was considerable variability in how outcomes were reported across studies in terms of units reported with some studies failing to report values for all timepoints, limiting the comparability between studies. The generalizability of findings is limited by the over-representation of studies in predominantly female, full time undergraduate student populations.

### Strengths and limitations of the review

Strengths of this review include the use of comprehensive search and screening strategies in the identification of relevant studies, two independent reviewers at each stage of the review, the use of the Cochrane Collaboration Tool for assessing risk of bias. In terms of limitations, restricting to studies published in English may have excluded relevant studies and may limit the generalisability of the review findings. It is also important to note that despite following a previously utilised methodology, certain methodological decisions (e.g. definition of effective, effectiveness ratio etc.) may have impacted the review results and only summary of the evidence. As such, the utilisation of different methodologies may result in different results and conclusions.

## Conclusions

There is limited evidence regarding the efficacy of health behaviour interventions in improving both health behaviour and mental health outcomes of university students. There is also insufficient evidence regarding intervention impact, intervention characteristics associated with improved outcomes and economic evaluation to guide the implementation of these interventions in the university setting. To further progress the research field and ensure that future systematic reviews can best inform the implementation of health behaviour interventions in the university setting, we propose the following recommendations for future research:

- Explore the comparison of single behaviour interventions to multi-behaviour interventions that target a range of health behaviours in improving both health behaviour and mental health outcomes.
- Investigating the efficacy of interventions targeting sleep behaviour change with a focus on exploring the effects of various facets of sleep which may impact changes in mental health status.
- Comprehensively reporting details of recruitment, retention and fidelity by reporting key information (i.e. definition, analysis strategy, reference of measure, data provider, level of data collected, type of data collected, frequency of outcome measurement, and unit of observation and analysis for each implementation outcome) within the main paper, protocol paper or supplementary information to enhance intervention validity and promote knowledge synthesis across interventions and studies Further exploring interventions characteristics associated with improved outcomes with a specific focus on those suggested to be more effective in this review.
- Conducting an economic evaluation of interventions to allow the maximisation of investments for mental health care within the university setting, including comparison to currently offered approaches to treatment and prevention of mental ill health in the university sector.

## Electronic supplementary material

Below is the link to the electronic supplementary material.

Supplementary Material 1

## Data Availability

All data generated or analysed during this study are included in this published article and its supplementary information files.

## Abbreviations

RCT: Randomised Controlled Trial
CBT: Cognitive Behavioural Therapy
PIPE: Penetration, Implementation, Participation, Effect
TIDieR: Template for Intervention Description & Replication
CBT-I: Cognitive Behavioural Therapy Interventions
ER: Effectiveness Ratio
