# Supplementary material for: Health behaviour interventions to improve mental health outcomes for students in the university setting: a systematic review of randomised controlled trials
Source: Int J Behav Nutr Phys Act. 2025 Mar 11;22:32. doi: 10.1186/s12966-025-01718-7 (PMC11900387; doi:10.1186/s12966-025-01718-7)
Supplement: Supplementary file 1 — Supplementary Material 1 [file 12966_2025_1718_MOESM1_ESM.docx]

**Additional information: Health Behaviour Interventions to Improve Mental Health Outcomes for Students in the University Setting: A Systematic Review of Randomised Controlled Trials**

Sandya Streram, Tracy Burrows, Mitch J Duncan, Melinda Hutchesson

**Contents**

| ***Supporting information item*** | ***Page*** |
| --- | --- |
| Table A1: Search Terms by database | 2 |
| Table A2: Detailed study characteristics of the included studies | 13 |
| Table A3: PRISMA 2020 Checklist | 21 |
| Table A4: PRISMA 2020 for Abstracts Checklist | 25 |

**Table A1:** Search Terms by database

*Ovid MEDLINE(R) and Epub Ahead of Print, In-Process, In-Data-Review & Other Non-Indexed Citations and Daily*

| **#** | **Searches** |
| --- | --- |
| 1 | Universities/ |
| 2 | (universit* or college* or polytechnic* or educational institution*).tw,kw. |
| 3 | 1 or 2 |
| 4 | Energy Intake/ or Feeding Behavior/ |
| 5 | ((energy or micronutrient* or macronutrient* or food or fruit* or vegetable* or fat* or sugar*) adj5 (intake* or consum*)).mp. |
| 6 | ((energy or diet*) adj5 (quality or pattern* or behavio* or habit* or intake*)).mp. |
| 7 | physical activit*.mp. |
| 8 | Exercise/ |
| 9 | aerobic.mp. |
| 10 | Sedentary Behavior/ |
| 11 | ((sedentary or sitting) adj5 (time* or behavio*)).mp. |
| 12 | Screen Time/ |
| 13 | (alcohol* adj5 (intake* or consum* or use*)).mp. |
| 14 | Sleep/ |
| 15 | alert*.mp. |
| 16 | wakeful*.mp. |
| 17 | drows*.mp. |
| 18 | (sleep* adj5 (quality or tim*)).mp. |
| 19 | Cigarette Smoking/ or Smoking/ or Electronic Nicotine Delivery Systems/ or vaping/ |
| 20 | "Tobacco Use"/ |
| 21 | ((smok* or tobacco or cigar* or e-cig* or ecig* or electronic cig* or electronic nicotine or vape or vaping or e-nicotine) adj5 use*).mp. |
| 22 | Alcohol Drinking/ |
| 23 | ((drug* or substance* or cannabis or hallucinogen* or phencyclidine or benzodiazepine or inhal* or opioid* or sedative* or hypnotic or anxiolytic* or stimulant* or amphetamine* or cocaine or marijuana) adj5 (use* or consum* or intake*)).mp. |
| 24 | ((health or healthy or lifestyle) adj3 (behavio* or factor*)).mp. |
| 25 | 4 or 5 or 6 or 7 or 8 or 9 or 10 or 11 or 12 or 13 or 14 or 15 or 16 or 17 or 18 or 19 or 20 or 21 or 22 or 23 or 24 |
| 26 | Randomized Controlled Trial/ |
| 27 | controlled clinical trial/ |
| 28 | Clinical Trial/ |
| 29 | random*.tw. |
| 30 | (control adj group*).tw. |
| 31 | 26 or 27 or 28 or 29 or 30 |
| 32 | Students/ |
| 33 | (student* or undergraduate* or postgraduate* or graduate* or baccalaureate or bachelor*).mp. |
| 34 | 32 or 33 |
| 35 | 3 and 25 and 31 and 34 |
| 36 | animals/ not humans/ |
| 37 | 35 not 36 |
| 38 | academic dissertation/ or meeting abstract/ or news/ or newspaper article/ or unpublished work/ |
| 39 | 37 not 38 |
| **40** | **limit 39 to (english language and yr="2012 -Current")** |

*Embase*

| **#** | **Searches** |
| --- | --- |
| 1 | university/ |
| 2 | (universit* or college* or polytechnic* or educational institution*).tw. |
| 3 | 1 or 2 |
| 4 | caloric intake/ |
| 5 | feeding behavior/ |
| 6 | ((energy or micronutrient* or macronutrient* or food or fruit* or vegetable* or fat* or sugar*) adj5 (intake* or consum*)).tw. |
| 7 | ((energy or diet*) adj5 (quality or pattern* or behavio* or habit* or intake*)).tw. |
| 8 | physical activity/ |
| 9 | exercise/ |
| 10 | aerobic exercise/ |
| 11 | sedentary lifestyle/ |
| 12 | ((sedentary or sitting) adj5 (time* or behavio*)).tw. |
| 13 | screen time/ |
| 14 | (alcohol* adj5 (intake* or consum* or use*)).tw. |
| 15 | sleep/ |
| 16 | alert*.tw. |
| 17 | wakeful*.tw. |
| 18 | drows*.tw. |
| 19 | (sleep* adj5 (quality or tim*)).tw. |
| 20 | smoking/ or cigarette smoking/ |
| 21 | "tobacco use"/ |
| 22 | ((smok* or tobacco or cigar* or e-cig* or ecig* or electronic cig* or electronic nicotine or vape or vaping or e-nicotine) adj5 use*).mp. |
| 23 | drinking behavior/ |
| 24 | ((drug* or substance* or cannabis or hallucinogen* or phencyclidine or benzodiazepine or inhal* or opioid* or sedative* or hypnotic or anxiolytic* or stimulant* or amphetamine* or cocaine or marijuana) adj5 (use* or consum* or intake*)).tw. |
| 25 | ((health or healthy or lifestyle) adj3 (behavio* or factor*)).mp. |
| 26 | 4 or 5 or 6 or 7 or 8 or 9 or 10 or 11 or 12 or 13 or 14 or 15 or 16 or 17 or 18 or 19 or 20 or 21 or 22 or 23 or 24 or 25 |
| 27 | randomized controlled trial/ |
| 28 | controlled clinical trial/ |
| 29 | random*.tw. |
| 30 | (control adj group*).tw. |
| 31 | 27 or 28 or 29 or 30 |
| 32 | college student/ or graduate student/ or PhD student/ or postgraduate student/ or university student/ or undergraduate student/ |
| 33 | (student* or undergraduate* or postgraduate* or graduate* or baccalaureate or bachelor*).tw. |
| 34 | 32 or 33 |
| 35 | 3 and 26 and 31 and 34 |
| 36 | abstract report/ or conference paper/ or erratum/ |
| 37 | 35 not 36 |
| 38 | animal/ not human/ |
| 39 | 37 not 38 |
| **40** | **limit 39 to (english language and yr="2012 -Current")** |

*APA PsycInfo*

| **#** | **Searches** |
| --- | --- |
| 1 | colleges/ |
| 2 | (universit* or college* or polytechnic* or educational institution*).mp. |
| 3 | 1 or 2 |
| 4 | Food Intake/ |
| 5 | Eating Behavior/ |
| 6 | ((energy or micronutrient* or macronutrient* or food or fruit* or vegetable* or fat* or sugar*) adj5 (intake* or consum*)).mp. |
| 7 | ((energy or diet*) adj5 (quality or pattern* or behavio* or habit* or intake*)).mp. |
| 8 | physical activit*.mp. |
| 9 | Exercise/ or Aerobic Exercise/ |
| 10 | Sedentary Behavior/ |
| 11 | ((sedentary or sitting) adj5 (time* or behavio*)).mp. |
| 12 | Screen Time/ |
| 13 | (alcohol* adj5 (intake* or consum* or use*)).mp. |
| 14 | Sleep/ |
| 15 | alert*.mp. |
| 16 | wakeful*.mp. |
| 17 | drows*.mp. |
| 18 | (sleep* adj5 (quality or tim*)).mp. |
| 19 | Tobacco Smoking/ |
| 20 | ((smok* or tobacco or cigar* or e-cig* or ecig* or electronic cig* or electronic nicotine or vape or vaping or e-nicotine) adj5 use*).mp. |
| 21 | Alcohol Drinking Patterns/ |
| 22 | ((drug* or substance* or cannabis or hallucinogen* or phencyclidine or benzodiazepine or inhal* or opioid* or sedative* or hypnotic or anxiolytic* or stimulant* or amphetamine* or cocaine or marijuana) adj5 (use* or consum* or intake*)).mp. |
| 23 | ((health or healthy or lifestyle) adj3 (behavio* or factor*)).mp. |
| 24 | 4 or 5 or 6 or 7 or 8 or 9 or 10 or 11 or 12 or 13 or 14 or 15 or 16 or 17 or 18 or 19 or 20 or 21 or 22 or 23 |
| 25 | Randomized Controlled Trials/ |
| 26 | clinical trials/ |
| 27 | randomized clinical trials/ |
| 28 | random*.tw. |
| 29 | (control adj group*).tw. |
| 30 | 25 or 26 or 27 or 28 or 29 |
| 31 | Graduate Students/ or College Students/ or Postgraduate Students/ |
| 32 | (student* or undergraduate* or postgraduate* or graduate* or baccalaureate or bachelor*).mp. |
| 33 | 31 or 32 |
| 34 | 3 and 24 and 30 and 33 |
| 35 | limit 34 to dissertation |
| 36 | 34 not 35 |
| **37** | **limit 36 to (human and english language and yr="2012 -Current")** |

*CINAHL*

| **#** | **Query** |
| --- | --- |
| S1 | (MH "Colleges and Universities") |
| S2 | TI ( (universit* or college* or polytechnic* or educational institution*) ) OR AB ( (universit* or college* or polytechnic* or educational institution*) ) |
| S3 | S1 OR S2 |
| S4 | (MH "Energy Intake") |
| S5 | (MH "Eating Behavior") |
| S6 | TI ( ((energy or micronutrient* or macronutrient* or food or fruit* or vegetable* or fat* or sugar*) n5 (intake* or consum*)) ) OR AB ( ((energy or micronutrient* or macronutrient* or food or fruit* or vegetable* or fat* or sugar*) n5 (intake* or consum*)) ) |
| S7 | TI ( ((energy or diet*) n5 (quality or pattern* or behavio* or habit* or intake*)) ) OR AB ( ((energy or diet*) n5 (quality or pattern* or behavio* or habit* or intake*)) ) |
| S8 | "physical activit*" |
| S9 | (MH "Exercise") |
| S10 | (MH "Aerobic Exercises") |
| S11 | TI ( ((sedentary or sitting) n5 (time* or behavio*)) ) OR AB ( ((sedentary or sitting) n5 (time* or behavio*)) ) |
| S12 | (MH "Screen Time") |
| S13 | TI ( (alcohol* n5 (intake* or consum* or use*)) ) OR AB ( (alcohol* n5 (intake* or consum* or use*)) ) |
| S14 | (MH "Sleep") |
| S15 | "alert*" |
| S16 | (MH "Wakefulness") |
| S17 | "drows*" |
| S18 | TI ( (sleep* n5 (quality or tim*)) ) OR AB ( (sleep* n5 (quality or tim*)) ) |
| S19 | (MH "Smoking") OR ((health or healthy or lifestyle) n3 (behavio* or factor*)) |
| S20 | TI ((smok* or tobacco or cigar* or e-cig* or ecig* or electronic cig* or electronic nicotine or vape or vaping or e-nicotine) N5 use*) OR AB ((smok* or tobacco or cigar* or e-cig* or ecig* or electronic cig* or electronic nicotine or vape or vaping or e-nicotine) N5 use*) |
| S21 | (MH "Alcohol Drinking") OR (MH "Alcohol Drinking in College") OR (MH "Drinking Behavior") |
| S22 | TI ( ((drug* or substance* or cannabis or hallucinogen* or phencyclidine or benzodiazepine or inhal* or opioid* or sedative* or hypnotic or anxiolytic* or stimulant* or amphetamine* or cocaine or marijuana) n5 (use* or consum* or intake*)))) ) OR AB ( ((drug* or substance* or cannabis or hallucinogen* or phencyclidine or benzodiazepine or inhal* or opioid* or sedative* or hypnotic or anxiolytic* or stimulant* or amphetamine* or cocaine or marijuana) n5 (use* or consum* or intake*)))) ) |
| S23 | S4 OR S5 OR S6 OR S7 OR S8 OR S9 OR S10 OR S11 OR S12 OR S13 OR S14 OR S15 OR S16 OR S17 OR S18 OR S19 OR S20 OR S21 OR S22 |
| S24 | (MH "Randomized Controlled Trials") |
| S25 | (MH "Clinical Trials") |
| S26 | TI random* OR AB random* |
| S27 | TI (control n1 group*) OR AB (control n1 group*) |
| S28 | S24 OR S25 OR S26 OR S27 |
| S29 | (MH "Students, College") OR (MH "Students, Graduate+") OR (MH "Students, Undergraduate") |
| S30 | (student* or undergraduate* or postgraduate* or graduate* or baccalaureate or bachelor*) |
| S31 | S29 OR S30 |
| S32 | S3 AND S23 AND S28 AND S31 |
| S33 | (MH "Newsletters") OR (MH "Newspapers") OR (MH "Theses and Dissertations") OR (MH "Gray Literature") |
| **S34** | **S32 NOT S33 restricted to English and 2012+** |

*CENTRAL*

| **ID** | **Search** |
| --- | --- |
| #1 | MeSH descriptor: [Universities] this term only |
| #2 | (universit* or college* or polytechnic* or "educational institution*"):ti,ab |
| #3 | {OR #1-#2} |
| #4 | MeSH descriptor: [Energy Intake] this term only |
| #5 | MeSH descriptor: [Feeding Behavior] this term only |
| #6 | ((energy or micronutrient* or macronutrient* or food or fruit* or vegetable* or fat* or sugar*) near/5 (intake* or consum*)):ti,ab |
| #7 | ((energy or diet*) near/5 (quality or pattern* or behavio* or habit* or intake*)):ti,ab |
| #8 | physical NEAR activit*:ti,ab |
| #9 | MeSH descriptor: [Exercise] this term only |
| #10 | aerobic:ti,ab |
| #11 | MeSH descriptor: [Sedentary Behavior] this term only |
| #12 | ((sedentary or sitting) near/5 (time* or behavio*)):ti,ab |
| #13 | MeSH descriptor: [Screen Time] this term only |
| #14 | (alcohol* near/5 (intake* or consum* or use*)) |
| #15 | MeSH descriptor: [Sleep] this term only |
| #16 | alert*:ti,ab |
| #17 | wakeful*:ti,ab |
| #18 | drows*:ti,ab |
| #19 | (sleep* near/5 (quality or tim*)):ti,ab |
| #20 | MeSH descriptor: [Cigarette Smoking] this term only |
| #21 | MeSH descriptor: [Tobacco Use] this term only |
| #22 | ((smok* or tobacco or cigar* or e-cig* or ecig* or electronic cig* or electronic nicotine or vape or vaping or e-nicotine) NEAR/5 use*):ti,ab |
| #23 | MeSH descriptor: [Alcohol Drinking] this term only |
| #24 | ((drug* or substance* or cannabis or hallucinogen* or phencyclidine or benzodiazepine or inhal* or opioid* or sedative* or hypnotic or anxiolytic* or stimulant* or amphetamine* or cocaine or marijuana) near/5 (use* or consum* or intake*)) or ((health or healthy or lifestyle) near/3 (behavio* or factor*)):ti,ab |
| #25 | {OR #4-#24} |
| #26 | MeSH descriptor: [Students] this term only |
| #27 | (student* or undergraduate* or postgraduate* or graduate* or baccalaureate or bachelor*):ti,ab |
| #28 | {OR #26-#27} |
| **#29** | **{AND #3, #25, #28} with Publication Year from 2012 to 2022, in Trials** |

*ERIC*

ab("energy intake" OR "feeding behavio*" OR energy OR micronutrient* OR macronutrient* OR food OR fruit* OR vegetable* OR fat* OR sugar* OR diet* OR "physical activit*" OR exercise OR aerobic OR "Sedentary Behavio*" OR sitting OR "Screen Time" OR alcohol* OR sleep OR alert* OR wakeful* OR drows* OR smok* OR "e-cig*" OR ecig* OR "electronic cig*" OR "electronic nicotine" OR vape OR vaping OR "e-nicotine" OR drug* OR substance* OR cannabis OR hallucinogen* OR phencyclidine OR benzodiazepine OR inhal* OR opioid* OR sedative* OR hypnotic OR anxiolytic* OR stimulant* OR amphetamine* OR cocaine OR marijuana OR lifestyle OR "life style*") AND ab(universit* OR college* OR polytechnic* OR "educational institution*") AND ab(random* OR trial* OR group*) AND ab(student* OR undergraduate* OR postgraduate* OR graduate* OR baccalaureate OR bachelor*)

NOT (Reports AND Dissertations & Theses AND Encyclopedias & Reference Works AND Speeches & Presentations AND Books AND Conference Papers & Proceedings AND Other Sources AND Government & Official Publications)

2012-01-01 - 2022-06-28

Higher Education OR Postsecondary Education OR Two Year Colleges

*Education Research Complete*

| **#** | **Query** |
| --- | --- |
| S1 | TI ( ("energy intake" OR "feeding behavio*" OR energy OR micronutrient* OR macronutrient* OR food OR fruit* OR vegetable* OR fat* OR sugar* OR diet* OR "physical activit*" OR exercise OR aerobic OR "Sedentary Behavio*" OR sitting OR "Screen Time" OR alcohol* OR sleep OR alert* OR wakeful* OR drows* OR smok* OR "e-cig*" OR ecig* OR "electronic cig*" OR "electronic nicotine" OR vape OR vaping OR "e-nicotine" OR drug* OR substance* OR cannabis OR hallucinogen* OR phencyclidine OR benzodiazepine OR inhal* OR opioid* OR sedative* OR hypnotic OR anxiolytic* OR stimulant* OR amphetamine* OR cocaine OR marijuana OR lifestyle OR "life style*") ) OR AB ( ("energy intake" OR "feeding behavio*" OR energy OR micronutrient* OR macronutrient* OR food OR fruit* OR vegetable* OR fat* OR sugar* OR diet* OR "physical activit*" OR exercise OR aerobic OR "Sedentary Behavio*" OR sitting OR "Screen Time" OR alcohol* OR sleep OR alert* OR wakeful* OR drows* OR smok* OR "e-cig*" OR ecig* OR "electronic cig*" OR "electronic nicotine" OR vape OR vaping OR "e-nicotine" OR drug* OR substance* OR cannabis OR hallucinogen* OR phencyclidine OR benzodiazepine OR inhal* OR opioid* OR sedative* OR hypnotic OR anxiolytic* OR stimulant* OR amphetamine* OR cocaine OR marijuana OR lifestyle OR "life style*") ) |
| S2 | TI ( (universit* OR college* OR polytechnic* OR "educational institution*") ) OR AB ( (universit* OR college* OR polytechnic* OR "educational institution*") ) |
| S3 | TI ( (random* OR trial* OR group*) ) AND AB ( (random* OR trial* OR group*) ) |
| S4 | TI ( (student* OR undergraduate* OR postgraduate* OR graduate* OR baccalaureate OR bachelor*) ) OR AB ( (student* OR undergraduate* OR postgraduate* OR graduate* OR baccalaureate OR bachelor*) ) |
| **S5** | **S1 AND S2 AND S3 AND S4 Limited to 2012+** |

*SCOPUS*

TITLE-ABS ( ( "energy intake" OR "feeding behavio*" OR energy OR micronutrient* OR macronutrient* OR food OR fruit* OR vegetable* OR fat* OR sugar* OR diet* OR "physical activit*" OR exercise OR aerobic OR "Sedentary Behavio*" OR sitting OR "Screen Time" OR alcohol* OR sleep OR alert* OR wakeful* OR drows* OR smok* OR "e-cig*" OR ecig* OR "electronic cig*" OR "electronic nicotine" OR vape OR vaping OR "e-nicotine" OR drug* OR substance* OR cannabis OR hallucinogen* OR phencyclidine OR benzodiazepine OR inhal* OR opioid* OR sedative* OR hypnotic OR anxiolytic* OR stimulant* OR amphetamine* OR cocaine OR marijuana OR lifestyle OR "life style*" ) ) AND TITLE-ABS ( universit* OR college* OR polytechnic* OR "educational institution*" ) AND TITLE ( random* OR trial* OR group* ) AND TITLE-ABS ( student* OR undergraduate* OR postgraduate* OR graduate* OR baccalaureate OR bachelor ) AND ( LIMIT-TO ( PUBYEAR , 2022 ) OR LIMIT-TO ( PUBYEAR , 2021 ) OR LIMIT-TO ( PUBYEAR , 2020 ) OR LIMIT-TO ( PUBYEAR , 2019 ) OR LIMIT-TO ( PUBYEAR , 2018 ) OR LIMIT-TO ( PUBYEAR , 2017 ) OR LIMIT-TO ( PUBYEAR , 2016 ) OR LIMIT-TO ( PUBYEAR , 2015 ) OR LIMIT-TO ( PUBYEAR , 2014 ) OR LIMIT-TO ( PUBYEAR , 2013 ) OR LIMIT-TO ( PUBYEAR , 2012 ) )

**Table A2:** Detailed characteristics of included studies.

| **First author, year, citation** | **Country** | **Length of follow up** | **Study participants (age range/mean; % female )** | **N at baseline** | **Details of exposure measures** | | | | | | | | **Details of mental health outcome/s** | **Significant findings** |
| --- | --- | --- | --- | --- | --- | --- | --- | --- | --- | --- | --- | --- | --- | --- |
|  |  |  |  |  | **Dietary intake** | **Physical activity** | **Sedentary behaviour** | **Alcohol intake** | **Sleep** | **Smoking** | **Illicit Drug use** | **Exposure measured more than once (Y/N)** |  |  |
| Duan,  2017 | China | 8 and 12 weeks | 17-24 yrs/19 yrs ;71%; | 493 | Fruit and vegetable intake | Amount of time spent on moderate – vigorous activity |  |  |  |  |  | Y | Quality of Life (Hong Kong version of the World Health Organization’s Quality of Life-BREF questionnaire), Depression (Chinese version of the Center  for Epidemiologic Studies Depression (CES-D) scale) | Increased fruit and vegetable intake: No impact on mental health outcomes |
| Spanhel, 2022 | Germany | 4 and 12 weeks | 20-42 yrs/ 27 yrs; 49% | 81 |  |  |  |  | Insomnia severity (Pittsburgh Sleep Quality Index) |  |  | Y | Depressive symptoms. (Patient Health Questionnaire-8) Anxiety symptoms ( General Anxiety Disorder-7), Perceived stress (Perceived Stress Scale -4) | Decreased insomnia severity: decreased depressive symptoms |
| Taylor, 2014 | US | 6 and 13 weeks | 18-27 yrs/ yrs; 59% | 34 |  |  |  |  | Insomnia severity, sleep efficiency, sleep and daytime functioning, actigraphy |  | Marijuana use (Marijuana Problem Scale) | Y | Quality of life (Quality of Life Enjoyment and Satisfaction Questionnaire) , Depressive symptoms (Quick Inventory of Depressive Symptomology) | Improved sleep efficiency, daytime functioning and insomnia severity |
| Murphy, 2012 | US | 4 and 20/30 weeks | 18-21 yrs/ 19 yrs; 50% | 82 |  |  |  | Number of drinks consumed daily (Daily Drinking Questionnaire) |  |  |  | Y | Depression, anxiety, stress (DASS scale) | NA |
| Shuai, 2022 | UK | 2 weeks | 18-25 yrs/ 20 yrs; 87% | 76 |  |  |  | Alcohol units consumed over the past 14 days (Daily Drinking Questionnaire) |  |  |  | N | Depressive symptoms ( Patient Health Questionnaire), Anxiety ( Generalised Anxiety Disorder) | Decreased depressive symptoms |
| Okajima, 2022 | Japan | 8 weeks | N/N: 67% | 48 |  |  |  |  | Insomnia severity, sleep hygiene practices, pre-sleep arousal |  |  | N | Depression, anxiety, stress | Improved insomnia severity, sleep hygiene and pre-sleep arousal; reduced depression and anxiety |
| Paulus, 2021 | US | 1,4 and 13 weeks | ≥18 yrs/ 22 yrs; 77% | 125 |  |  |  | Hazardous alcohol use (AUDIT-C) |  |  |  | Y | Anxiety sensitivity (Anxiety Sensitivity Index-3 :ASI-3) & Anxiety and Depression ( Mood Anxiety Symptom Questionnaire short form :MASQ) | Reduced hazardous alcohol consumption at 3 months; reduced anxiety sensitivity |
| Sandrick, 2017 | US | 8 weeks | 18-30 yrs/ 19 yrs; 68% | 60 | Diet quality (FFQ- Rate Your Plate) | Amount of exercise completed/ METS expended (IPAQ) |  |  | Sleep quality (PSQI), daytime sleepiness (ESS) |  |  | N | Perceived stress (PSS-14) | Increased physical activity |
| Huberty, 2019 | US | 8 and 12 weeks | ≥18 yrs/ 20 yrs; 79% | 109 | Eaten five servings of fruits or vegetables on most of the past 7 days (Youth Risk Behavior Surveillance (YRBS) survey) | Engaged in at least 150 minutes of physical activity during the past 7 days (Youth Risk Behavior Surveillance) |  | Alcohol consumption in past 7 days ie, engaged in binge drinking (Youth Risk Behavior Surveillance (YRBS) survey) |  |  |  | Y | Perceived stress (PSS) | Decreased perceived stress |
| Greene, 2012 | US | 13 and 65 weeks | 18-24 yrs/ 19 yrs; 63% | 1689 | Fruit and veg intake (cups per day) - Two-Item Screener and the National Cancer  Institute (NCI) Fruit and Vegetable Screener | Metabolic equivalent minutes per week (MET-min/wk) - IPAQ |  |  |  |  |  | Y | Emotional problems and stress (General Health Questionnaire) | Increased fruit and vegetable intake: Decreased emotional problems and stress |
| Hahn, 2021 | US | 4 weeks | ≥18 yrs/ 20 yrs; 100% | 200 | Number of times consumed per week - breakfast, intake of fruits, vegetables, combined fruits and vegetables, 100% fruit juice, soda, diet soda, energy drinks , sports drinks , low-calorie sports drinks, water, and sparkling water - YRBS survey | Amount of total exercise in past week - YRSB survey |  |  |  |  |  | N | Depressive symptoms (Center for Epidemiologic Studies Depression Scale Revised), State Anxiety ( State-Trait Anxiety Inventory) | NA |
| Buckner, 2020 | US | 2 weeks | ≥18 yrs/ 19 yrs; 83% |  |  |  |  |  |  |  | Cannabis use frequency in past 2 weeks- Timeline Follow Back (TLFB) | N | Social anxiety (SIAS-S 20-item measure), Emotions (Positive and negative affect scale) | Decreased cannabis use: decreased social anxiety |
| Pengpid,2013 | South Africa | 26 and 48 weeks | ≥18 yrs/ 22 yrs; 13% | 152 |  |  |  | Alcohol consumption levels, heavy episodic drinking -Alcohol Disorder Identification Test (AUDIT) |  | Tobacco use in past month | Cannabis use in past month | Y | Depressive symptoms ( Centers for Epidemiologic Studies Depression Scale (CES-D)) , PTSD | Decreased alcohol consumption, |
| Duan, 2022 | China | 4,8 and 12 weeks | ≥18 yrs/ 20 yrs; 58% | 565 | Fruit and vegetable consumption | Weekly MET-min of total PA - IPAQ |  |  |  |  |  | Y | Depression – (Chinese version of the  Centre for Epidemiologic Studies Depression–10 Scale) | Increased fruit and vegetable consumption |
| Sharp, 2016 | Canada | 12 weeks | ≥17 yrs/ 18 yrs; 53% | 184 |  | Average mins per week of strenous, moderate and mild physical activity completed in last 7 days - modified Godin Leisure-Time Exercise Questionnaire (GLTEQ) |  |  |  |  |  | N | Psychological wellbeing (GHQ-12) | NA |
| Freeman, 2017 | UK | 3, 10 and 22 weeks | ≥18 yrs/ 25 yrs; 72% | 3755 |  |  |  |  | Insomnia (SCI-8, insomnia severity index) |  |  | Y | Affective symptoms – (PHQ-9/ GAD-7), Anxiety, depression, psychological welleing (Warwick–Edinburgh  Mental Wellbeing Scale) | Decreased insomnia severity: Decreased affective symptoms, anxiety, depression, increased psychological wellbeing |
| Kattelman, 2014 | US | 13 and 64 weeks | 18-24 yrs/ 19 yrs; 67% | 1639 | Fruit and vegetable daily intake (cups per day)- National Cancer Institute Fruit and Vegetable Screener(short form) , percentage of calories from dietary fat in past year National Cancer Institute Fat Screener (short form), amount of SSB consumed, serves of wholegrains consumed per day | Amount of physical intensity performed at 3 intensity levels - IPAQ |  |  | Hours of sleep - Behaviour risk factor survey |  |  | Y | Perceived stress (PSS-14) | Increased fruit and vegetable consumption, decreased fat intake, higher number of hours of sleep |
| Yang, 2020 | China | 30 weeks | 16-24 yrs/ 20 yrs; 46% | 532 | Sugar sweetened beverages and breakfast consumption | Number of days performed at least 30 mins of physical activity |  |  |  |  |  | N | Subjective wellbeing ( 5-item WHO wellbeing index (WHO-5)) | Decreased sugar sweetened beverage consumption, increased breakfast consumption, increased physical activity |
| Hershner, 2018 | US | 1 and 8 weeks | ≥18 yrs/ 22 yrs; 58% | 549 |  |  |  |  | Sleepiness (ESS) and sleep quality (PSQI) |  |  | Y | Depression (PHQ-9) | Increased sleep quality: decreased depression symptoms |
| Whatnall, 2019 | Australia | 13 weeks | 17-35 yrs/ 22 yrs; 73% | 124 | Diet quality - ARFS |  |  | Frequency of alcohol consumption and number of standard drinks usually consumed per drinking occasion - New South Wales Adult Population Health  Survey |  |  |  | N | Wellbeing (WHO-Five Well-Being Index) | NA |
| Murphy, 2019 | US | 4, 26,52 and 69 weeks | N/ 19 yrs; 61% | 393 |  |  |  | Total number of standard drinks they consumed each day - DDQ |  |  |  | Y | Symptoms of depression,  anxiety and stress | Decreased weekly drinking: Decreased anxiety and depression |
| Yan, 2023 | US | 8 weeks | ≥18 yrs/ 19 yrs; 81% | 52 |  | Weekly MET-min of total PA - IPAQ |  |  | Sleep quality (PSQI) |  |  | N | Positive affect and wellbeing, anxiety (Neuro-QOL Item Bank v1.0) | Increased vigorous physical activity |

**Table A3:** PRISMA 2020 Checklist

| **Section and Topic** | **Item #** | **Checklist item** | **Location where item is reported** |
| --- | --- | --- | --- |
| **TITLE** | | |  |
| Title | 1 | Identify the report as a systematic review. | Page 1 |
| **ABSTRACT** | | |  |
| Abstract | 2 | See the PRISMA 2020 for Abstracts checklist. | Page 2-3 |
| **INTRODUCTION** | | |  |
| Rationale | 3 | Describe the rationale for the review in the context of existing knowledge. | Page 3-5 |
| Objectives | 4 | Provide an explicit statement of the objective(s) or question(s) the review addresses. | Page 5 |
| **METHODS** | | |  |
| Eligibility criteria | 5 | Specify the inclusion and exclusion criteria for the review and how studies were grouped for the syntheses. | Page 6-7, Table 1 |
| Information sources | 6 | Specify all databases, registers, websites, organisations, reference lists and other sources searched or consulted to identify studies. Specify the date when each source was last searched or consulted. | Page 5 |
| Search strategy | 7 | Present the full search strategies for all databases, registers and websites, including any filters and limits used. | Additional File, Table A1 |
| Selection process | 8 | Specify the methods used to decide whether a study met the inclusion criteria of the review, including how many reviewers screened each record and each report retrieved, whether they worked independently, and if applicable, details of automation tools used in the process. | Page 6 |
| Data collection process | 9 | Specify the methods used to collect data from reports, including how many reviewers collected data from each report, whether they worked independently, any processes for obtaining or confirming data from study investigators, and if applicable, details of automation tools used in the process. | Page 7-8 |
| Data items | 10a | List and define all outcomes for which data were sought. Specify whether all results that were compatible with each outcome domain in each study were sought (e.g. for all measures, time points, analyses), and if not, the methods used to decide which results to collect. | Page 7- 8, Table 2 |
|  | 10b | List and define all other variables for which data were sought (e.g. participant and intervention characteristics, funding sources). Describe any assumptions made about any missing or unclear information. | Page 7- 8, Table 2 |
| Study risk of bias assessment | 11 | Specify the methods used to assess risk of bias in the included studies, including details of the tool(s) used, how many reviewers assessed each study and whether they worked independently, and if applicable, details of automation tools used in the process. | Page 7 |
| Effect measures | 12 | Specify for each outcome the effect measure(s) (e.g. risk ratio, mean difference) used in the synthesis or presentation of results. | Page 7- 8, Table 2 |
| Synthesis methods | 13a | Describe the processes used to decide which studies were eligible for each synthesis (e.g. tabulating the study intervention characteristics and comparing against the planned groups for each synthesis (item #5)). | Page 7- 8, Table 2 |
|  | 13b | Describe any methods required to prepare the data for presentation or synthesis, such as handling of missing summary statistics, or data conversions. | Table 2 |
|  | 13c | Describe any methods used to tabulate or visually display results of individual studies and syntheses. | Table 2 |
|  | 13d | Describe any methods used to synthesize results and provide a rationale for the choice(s). If meta-analysis was performed, describe the model(s), method(s) to identify the presence and extent of statistical heterogeneity, and software package(s) used. | Table 2 |
|  | 13e | Describe any methods used to explore possible causes of heterogeneity among study results (e.g. subgroup analysis, meta-regression). | NA |
|  | 13f | Describe any sensitivity analyses conducted to assess robustness of the synthesized results. | NA |
| Reporting bias assessment | 14 | Describe any methods used to assess risk of bias due to missing results in a synthesis (arising from reporting biases). | NA |
| Certainty assessment | 15 | Describe any methods used to assess certainty (or confidence) in the body of evidence for an outcome. | Table 2 |
| **RESULTS** | | |  |
| Study selection | 16a | Describe the results of the search and selection process, from the number of records identified in the search to the number of studies included in the review, ideally using a flow diagram. | Page 8-9 |
|  | 16b | Cite studies that might appear to meet the inclusion criteria, but which were excluded, and explain why they were excluded. | NA |
| Study characteristics | 17 | Cite each included study and present its characteristics. | Page 8-10, Table 1 |
| Risk of bias in studies | 18 | Present assessments of risk of bias for each included study. | Page 10-11 |
| Results of individual studies | 19 | For all outcomes, present, for each study: (a) summary statistics for each group (where appropriate) and (b) an effect estimate and its precision (e.g. confidence/credible interval), ideally using structured tables or plots. | Page 11- 17 |
| Results of syntheses | 20a | For each synthesis, briefly summarise the characteristics and risk of bias among contributing studies. | Page 11- 17 |
|  | 20b | Present results of all statistical syntheses conducted. If meta-analysis was done, present for each the summary estimate and its precision (e.g. confidence/credible interval) and measures of statistical heterogeneity. If comparing groups, describe the direction of the effect. | Page 11-17 |
|  | 20c | Present results of all investigations of possible causes of heterogeneity among study results. | Page 11-17 |
|  | 20d | Present results of all sensitivity analyses conducted to assess the robustness of the synthesized results. | NA |
| Reporting biases | 21 | Present assessments of risk of bias due to missing results (arising from reporting biases) for each synthesis assessed. | Page 10-11 |
| Certainty of evidence | 22 | Present assessments of certainty (or confidence) in the body of evidence for each outcome assessed. | Table 3-5 |
| **DISCUSSION** | | |  |
| Discussion | 23a | Provide a general interpretation of the results in the context of other evidence. | Page 18-23 |
|  | 23b | Discuss any limitations of the evidence included in the review. | Page 23 |
|  | 23c | Discuss any limitations of the review processes used. | Page 23-24 |
|  | 23d | Discuss implications of the results for practice, policy, and future research. | Page 24-25 |
| **OTHER INFORMATION** | | |  |
| Registration and protocol | 24a | Provide registration information for the review, including register name and registration number, or state that the review was not registered. | Page 5 |
|  | 24b | Indicate where the review protocol can be accessed, or state that a protocol was not prepared. | Page 5 |
|  | 24c | Describe and explain any amendments to information provided at registration or in the protocol. | NA |
| Support | 25 | Describe sources of financial or non-financial support for the review, and the role of the funders or sponsors in the review. | Page 26 |
| Competing interests | 26 | Declare any competing interests of review authors. | Page 25 |
| Availability of data, code and other materials | 27 | Report which of the following are publicly available and where they can be found: template data collection forms; data extracted from included studies; data used for all analyses; analytic code; any other materials used in the review. | Page 25 |

*From:*  Page MJ, McKenzie JE, Bossuyt PM, Boutron I, Hoffmann TC, Mulrow CD, et al. The PRISMA 2020 statement: an updated guideline for reporting systematic reviews. BMJ 2021;372:n71. doi: 10.1136/bmj.n71. This work is licensed under CC BY 4.0. To view a copy of this license, visit <https://creativecommons.org/licenses/by/4.0/>

**Table A4:** PRISMA 2020 for Abstracts Checklist

| **Section and Topic** | **Item #** | **Checklist item** | **Reported (Yes/No)** |
| --- | --- | --- | --- |
| **TITLE** | | |  |
| Title | 1 | Identify the report as a systematic review. | Yes |
| **BACKGROUND** | | |  |
| Objectives | 2 | Provide an explicit statement of the main objective(s) or question(s) the review addresses. | Yes |
| **METHODS** | | |  |
| Eligibility criteria | 3 | Specify the inclusion and exclusion criteria for the review. | Yes |
| Information sources | 4 | Specify the information sources (e.g. databases, registers) used to identify studies and the date when each was last searched. | Yes |
| Risk of bias | 5 | Specify the methods used to assess risk of bias in the included studies. | Yes |
| Synthesis of results | 6 | Specify the methods used to present and synthesise results. | No |
| **RESULTS** | | |  |
| Included studies | 7 | Give the total number of included studies and participants and summarise relevant characteristics of studies. | Yes |
| Synthesis of results | 8 | Present results for main outcomes, preferably indicating the number of included studies and participants for each. If meta-analysis was done, report the summary estimate and confidence/credible interval. If comparing groups, indicate the direction of the effect (i.e. which group is favoured). | Yes |
| **DISCUSSION** | | |  |
| Limitations of evidence | 9 | Provide a brief summary of the limitations of the evidence included in the review (e.g. study risk of bias, inconsistency and imprecision). | No |
| Interpretation | 10 | Provide a general interpretation of the results and important implications. | No |
| **OTHER** | | |  |
| Funding | 11 | Specify the primary source of funding for the review. | No |
| Registration | 12 | Provide the register name and registration number. | No |

*From:*  Page MJ, McKenzie JE, Bossuyt PM, Boutron I, Hoffmann TC, Mulrow CD, et al. The PRISMA 2020 statement: an updated guideline for reporting systematic reviews. BMJ 2021;372:n71. doi: 10.1136/bmj.n71. This work is licensed under CC BY 4.0. To view a copy of this license, visit <https://creativecommons.org/licenses/by/4.0/>
